# Lung cancer immunotherapy: progress, pitfalls, and promises

**DOI:** 10.1186/s12943-023-01740-y

**Published:** 2023-02-21

**Authors:** Aritraa Lahiri, Avik Maji, Pravin D. Potdar, Navneet Singh, Purvish Parikh, Bharti Bisht, Anubhab Mukherjee, Manash K. Paul

**Affiliations:** 1 Department of Biological Sciences, Indian Institute of Science Education and Research Kolkata, Mohanpur, Nadia, West Bengal 741246 India; 2 Department of Radiation Oncology, N. R. S. Medical College & Hospital, 138 A.J.C. Bose Road, Kolkata, 700014 India; 3 Department of Molecular Medicine and Stem Cell Biology, Jaslok Hospital and Research Centre, Mumbai, 400026 India; 4 Department of Pulmonary Medicine, Postgraduate Institute of Medical Education and Research, Chandigarh, 160012 India; 5 Department of Clinical Hematology, Mahatma Gandhi Medical College and Hospital, Jaipur, Rajasthan 302022 India; 6 Department of Medical Oncology, Tata Memorial Hospital, Mumbai, Maharashtra 400012 India; 7 Division of Thoracic Surgery, David Geffen School of Medicine, University of California Los Angeles, Los Angeles, CA 90095 USA; 8 Esperer Onco Nutrition Pvt Ltd, 4BA, 4Th Floor, B Wing, Gundecha Onclave, Khairani Road, Sakinaka, Andheri East, Mumbai, Maharashtra 400072 India; 9 Department of Pulmonary and Critical Care Medicine, David Geffen School of Medicine, University of California Los Angeles, Los Angeles, CA 90095 USA; 10 Department of Microbiology, Kasturba Medical College, Manipal Academy of Higher Education, Manipal, Karnataka 576104 India

**Keywords:** Lung Cancer, SCLC, NSCLC, Immunotherapy, Nanomedicine, Cancer Vaccine, Antibody, Adaptive cell therapy, CAR T therapy, TCR T therapy, TIL therapy, Immunomodulators

## Abstract

Lung cancer is the primary cause of mortality in the United States and around the globe. Therapeutic options for lung cancer treatment include surgery, radiation therapy, chemotherapy, and targeted drug therapy. Medical management is often associated with the development of treatment resistance leading to relapse. Immunotherapy is profoundly altering the approach to cancer treatment owing to its tolerable safety profile, sustained therapeutic response due to immunological memory generation, and effectiveness across a broad patient population. Different tumor-specific vaccination strategies are gaining ground in the treatment of lung cancer. Recent advances in adoptive cell therapy (CAR T, TCR, TIL), the associated clinical trials on lung cancer, and associated hurdles are discussed in this review. Recent trials on lung cancer patients (without a targetable oncogenic driver alteration) reveal significant and sustained responses when treated with programmed death-1/programmed death-ligand 1 (PD-1/PD-L1) checkpoint blockade immunotherapies. Accumulating evidence indicates that a loss of effective anti-tumor immunity is associated with lung tumor evolution. Therapeutic cancer vaccines combined with immune checkpoint inhibitors (ICI) can achieve better therapeutic effects. To this end, the present article encompasses a detailed overview of the recent developments in the immunotherapeutic landscape in targeting small cell lung cancer (SCLC) and non-small cell lung cancer (NSCLC). Additionally, the review also explores the implication of nanomedicine in lung cancer immunotherapy as well as the combinatorial application of traditional therapy along with immunotherapy regimens. Finally, ongoing clinical trials, significant obstacles, and the future outlook of this treatment strategy are also highlighted to boost further research in the field.

## Introduction

Globally, cancer incidence and death are rising, with lung cancer being the most commonly diagnosed form of cancer (11.6% of the total cases). In the United States, in 2022, there are expected to be ~ 236,740 new lung cancer cases, with ~ 130,180 human demise [1]. Lung cancer remains the leading cause of global cancer mortalities (18.4% of total cancer fatalities), causing significant societal burden and economic loss [1, 2]. Around 80% of lung cancer deaths are caused by smoking. Other risk factors for lung cancer include radon, asbestos, long-term and cumulative exposure to air pollution, especially polycyclic aromatic hydrocarbons (PAH) emissions, and personal or familial lung cancer history [3, 4]. Lung tumors are divided into two broad categories by the World Health Organization (WHO); non-small cell lung cancer (NSCLC), comprising 80–85% of all lung cancer cases, and small cell lung cancer (SCLC), constituting the other 15% incidences [5–7]. NSCLC can be further subcategorized into adenocarcinoma (LUAD), squamous cell carcinoma (LUSC), and large cell carcinoma (LCC). Each subcategory based on the molecular targetable genetic profile can be subcategorized into several types [8]. It turns out that the survival rates for metastatic lung cancer of both NSCLC and SCLC types are poor, with a 5-year survival of only about 4% [9, 10].

Although several anti-cancer strategies like surgery, chemotherapy, and irradiation are used to treat NSCLC and SCLC, there is an urgent need for effective strategies to cure or manage lung cancer, particularly late-stage cancers [11]. The prognosis of NSCLC is challenging due to the unavailability of a platform for early-stage diagnosis and the late appearance of symptoms in disease development, limiting treatment choices and survival [12]. Low-dose computed tomography (LDCT) is the gold standard for current lung cancer patient screening. So far, in the USA, only 5% of the 15 million high-risk individuals advised for screening have used LDCT. LDCT suffers from low early detection efficiency, false-positive detection, radiation hazard, and unavailability of resources for running an efficient CT-based screening program [13]. Though early detection increases the likelihood of tumor resection, treatment, and a successful outcome, the unavailability of an appropriate screening platform, metastatic nature, genetic heterogeneity, and minimal response to chemotherapy at late stages make lung cancer fatal [14]. However, chemotherapy and radiation are recommended (including neoadjuvant and/or adjuvant therapy) for locally advanced and metastatic cancers but have shown limited overall survival (OS) and toxic side effects. Targeted therapies along with chemotherapy have become standard therapies for NSCLC patients with actionable oncogenic alterations (driver mutations and fusions/rearrangements), resulting in increased progression-free survival (PFS) and the OS in several cases. Targeted therapies have differing side effect profiles compared to chemotherapy and may not necessarily have sustained treatment responses [15, 16].

SCLC is classified based on the extension of the disease into a limited disease SCLC (LD-SCLC) and an extensive disease SCLC (ED-SCLC). Although new chemotherapeutic agents are being continuously formulated, the prognosis remains poor due to aggressive progression, lack of early detection techniques, limited treatment options, and efficacy [16, 17]. For LD-SCLC, a standard strategy is chemotherapy (cisplatin or carboplatin with etoposide) combined with thoracic radiotherapy [18]. SCLC initially responds well to chemotherapy and radiation but often relapses, leading to poor survival. The median survival (MS) rate for this group of patients is approximately 7–12 months due to limited early detection modalities, dearth of tissue availability for clinical research, tumor genetic heterogeneity, and poor understanding of molecular mechanisms leading to rapid progression and therapeutic resistance [19, 20]. Clinical studies of new drugs and targeted molecular treatment for SCLC have shown limited, encouraging results [5, 21]. Hence, there is a pressing need for a new treatment modality with a persistent response.

Recent research has refined our understanding of the immune system's reaction to cancer and how to enhance it, leading to considerable improvements in cancer immunotherapy [22]. Immunotherapy possesses potential efficacy irrespective of the histology and driver mutational status, leading to sustained remission, especially for those patients who exhibit a response [23]. The goal of cancer immunotherapy is to elicit (or re-elicit) a cellular immune response, especially the T-cell-mediated tumor-specific antigen (TSA) and tumor-associated antigens (TAA)-directed cytotoxicity that can selectively destroy a tumor [24]. The immune-modulatory drugs can also counter cancer cells by increasing the concentration of tumor-specific antibodies, natural killer (NK) cells, dendritic cells (DCs), macrophages (MΦ), and cytokines in the blood plasma [25]. However, in the past few years, immunotherapy has been considered inapt for lung cancer due to minimal immune responses [26]. Lung cancer immunotherapy is challenging as the cells avoid immunosurveillance and reduce the overall immunological response by modulating the T-cell mediated cytotoxicity, secretion of immune-suppressive cytokines, and loss of major histocompatibility complex (MHC) expression [27]. Recent technical advances have helped determine the molecular granularity of lung cancer immunogenicity, and since then various types of immunotherapies have evolved for treating lung cancer. Immunotherapy treatment types include therapeutic vaccines, immune modulators, autologous cellular therapies, and monoclonal antibodies (mAbs) directed against checkpoint inhibitor signals associated with activated T-cells and/or with cancer cells. However, since each therapeutic approach has distinct advantages and disadvantages, combining multiple therapies or therapeutic strategies with immunotherapy is preferable [28]. The present article examines recent advances in lung cancer (NSCLC and SCLC) immunotherapy, continuing clinical studies of immunotherapeutic interventions, and future directions.

### NSCLC and immunotherapy

LUAD is the most prevalent NSCLC, especially in the USA, accounts for around 40% of all lung cancer, and occurs in smokers and non-smokers regardless of their age and sex [29]. LUAD arises from the glandular cells of the alveoli (tiny air sacs) and tends to occur in the peripheral regions of the lung [30]. Due to its slow development rate than other types of lung cancer, it is more likely to be detected before it metastasizes beyond the lungs [31, 32]. LUSC is the second most common type of lung cancer, accounting for 25–30% of all lung cancer occurrences. LUSC is connected with smoking more than any other kind of NSCLC and is characterized by recurring somatically altered genes and pathways linked to smoking [33, 34]. Tracheobronchial squamous cells, particularly the basal cells, often give birth to squamous cell lung tumors, which are found mostly in the central part of the lung (the major airways) but may also occur peripherally [33, 34]. The third type, LCC accounts for approximately 5–10% of lung cancers and are also associated with smoking [35]. LCC generally shows no evidence of squamous or glandular maturation and remain undifferentiated, and as a result, it is often diagnosed through the exclusion of other possibilities. They habitually begin from the central part of the lungs, spread quickly, sometimes invading nearby lymph nodes, have chest wall involvement, and metastasize to distant organs [35, 36]. The lung cancer staging project by the International Association for the study of Lung Cancer (IASLC) revealed that patients were more likely to survive if diagnosed and treated in the early pathological stage with an MS of 95 months for stage IA, 75 months for stage IB, 44 months for stage IIA, 29 months for stage IIB and 19 months for stage IIIA. Also, a considerable influence factor on OS was the subtype of tumor cells [83 months for Bronchoalveolar carcinoma (uncommon type of LUAD), 45 months for LUAD, 44 months for LUSC, 34 months for LCC, and 26 months for Adenosquamous carcinoma] [37]. However, with the recent awareness about smoking cessation and improvements in early diagnosis and treatment, mortality rates of lung cancer have steadily dropped during the last two decades [38]. Immunotherapy is one such treatment advancement that has impacted patient survival in lung cancer, especially NSCLC. In this regard, understanding and accumulation of know-how about the immune mechanisms, driver mutations, neoantigens, and oncogenic pathways involved in NSCLC have brought about more clarity regarding the heterogeneity of tumor, mutational burden, and tumor microenvironment (TME), which has aided in designing new immunotherapeutic tools for targetable mutations [39].

**Fig. 1:**
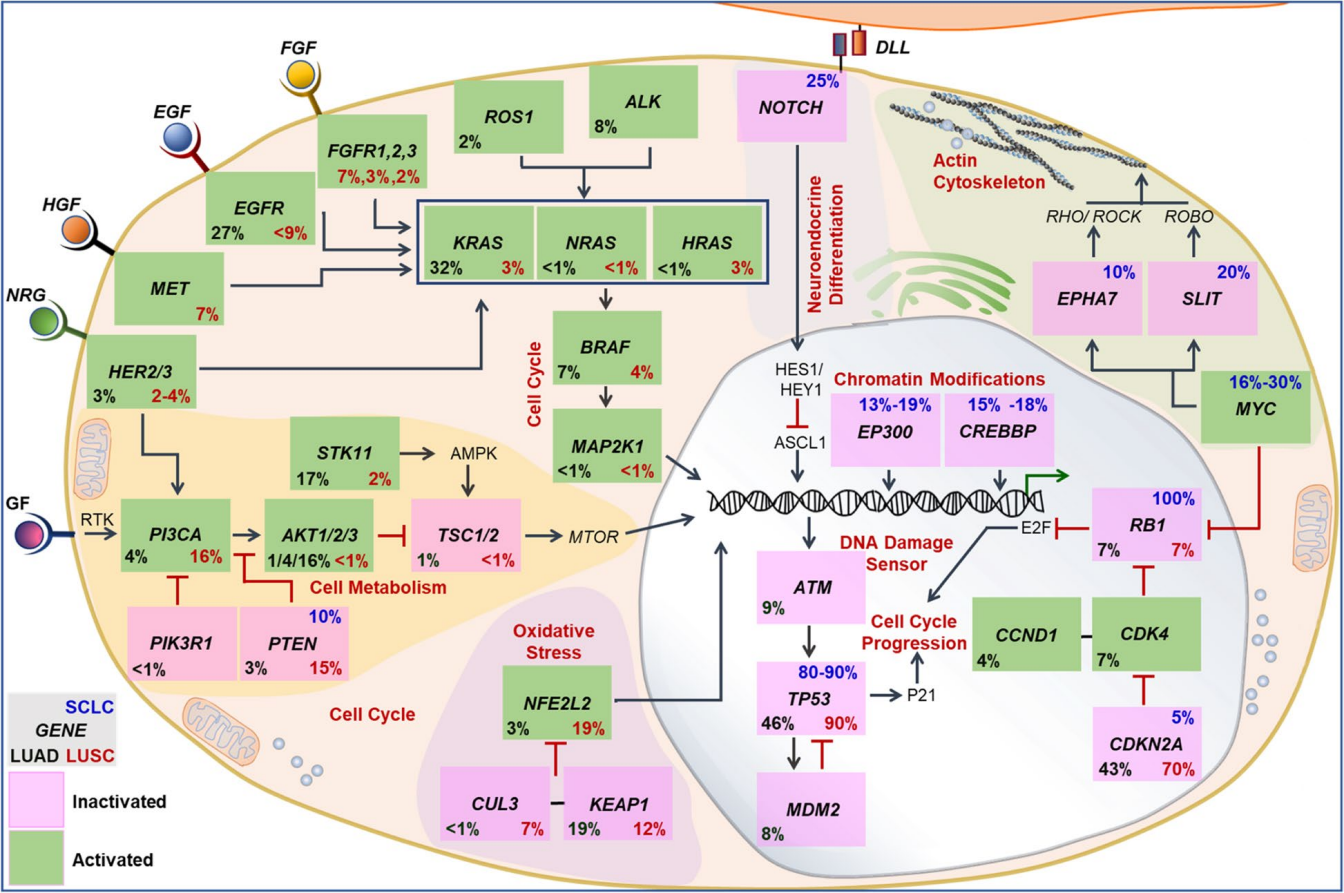
Genetic profiling of lung cancer (SCLC, LUAD, and LUSC) have shown changes in several oncogenes and tumor suppressor genes. Based on the total of somatic mutations, homozygous deletions, localized amplification, and substantial changes in gene expression, the values in each box represent the rates of genomic abnormalities. Other crucial proteins that mediate the pathways are also discussed. EGF, epidermal growth factor; FGF, fibroblast growth factor; GF, growth factor; DLL, deltalike; EGF, epidermal growth factor; FGF, fibroblast growth factor; GF, growth factor; HGF, hepatocyte growth factor; NRG, neuregulin; RTK, receptor tyrosine kinase. Figure reproduced with permission from Reference 39

**Fig. 2:**
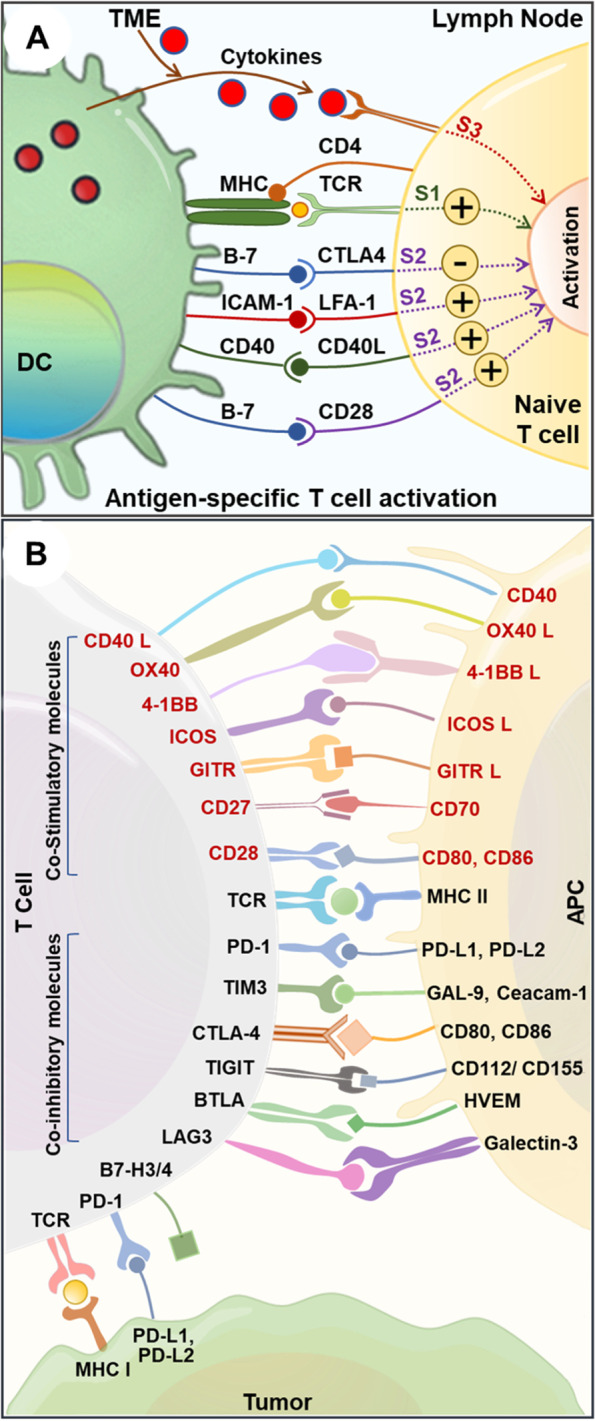
Immune interaction between T-cells, APCs, and cancer cells **A**. Schematic representing the mechanism of antigen-specific T cell activation. DCs play a crucial role in anti-tumor immunity due to their exceptional capacity to activate T cells following the central dogma of three signals. S1: Signal 1; S2: Signal 2; S3: Signal 3; TME: Tumor microenvironment; DC: Dendritic cell. **B**. Schematic showing immune interaction between T cell and APC; T cell with tumor cells. The T cell co-inhibitory and co-stimulatory molecules are shown in black and red fonts respectively. APC: Antigen presenting cell, L: Ligand

The lung cancer genome is characterized by a unique mutational landscape. Specific oncogenic mutations confer a dominant gain of function and recessive loss of function mutations in tumor suppressor genes. Somatic mutations, homozygous gene deletions, gene amplifications, gene translocations, and epigenetic silencing may cause genomic changes and alterations in specific pathways leading to the transformation of normal cells to premalignant cells and finally into lung tumors [40]. Lung cancer genetic profiling indicated considerable patient heterogeneity. It has been possible to identify several oncogenes and tumor suppressor genes (Fig. 1). KRAS, ALK, c-MET, RET, BRAF V600E, ROS1, NTRK, TP53, and ERBB2 (HER2) [40] are among the actionable genetic changes found in NSCLC. Genomic alteration-associated generation of tumor-specific antigens or neoantigens expressed by the premalignant/tumor cells, following antigen-presenting cell (APC)-mediated antigen presentation, can activate the T-cell specific adaptive antitumor immune response. The three-signal activation dogma governs classical T-cell activation. APCs display antigenic peptides on MHC I molecules to naïve T cells via their cognate T-cell receptor (TCR) (signal 1). A positive costimulatory signal, termed signal 2 (interaction of DC-specific CD80/86 and T cells-specific CD28 receptor), is essential for T cell activation. DCs further secrete pro-inflammatory cytokines (signal 3) to induce T cells toward antigen-specific antitumor response (Fig. 2A) [40]. These primed and activated effector T cells can infiltrate lung TME and effects tumor cell killing. When placed in the context of the inflammatory milieu of the tumor, signal 3 may help clarify the connection between chronic inflammation and lung cancer. The expression of the immune checkpoints is linked to several of the genetic modifications, like TP53, KRAS, and STK11 gene mutations. The details of the genetic underpinnings of lung cancer are covered in other reviews and chapters [40, 41].

Lung cancer immunotherapy, which aids the immune system in identifying and eliminating cancer cells, has gotten much attention lately. The nature of the interaction of tumor cells with the immune cells in the TME defines the antitumor response. Recent studies exemplify a dichotomous role of the immune cells during lung tumor evolution and can either promote an anti-tumor response or modulate an immunosuppressive (pro-tumorigenic) TME [42, 43]. TME is a complex signal interaction space surrounding the tumor, constituted by the endothelial cells, stromal fibroblast, mesenchymal cells, adipocytes, immune cells, and the extracellular matrix. The composition and the pathological significance of the tumor immune microenvironment (TIME) have been a topic of intense investigation in the last decade. The discovery of immune checkpoints (ICP), which are proteins produced by some immune cells (like T cells) and cancer cells, is an unprecedented moment in the history of immunotherapy. Under normal physiological conditions, the ICPs bind with their complementary companion proteins (receptor-ligand interaction), activate inhibitory signals, turn off T cell response, and.

thereby preventing an indiscriminate attack on healthy cells. They are crucial for self-tolerance, normal regulation of the immune system, and immunostasis. Tumor cells use this crucial regulatory process to their advantage and express ICP proteins to evade immune cell-mediated tumor cell death. Targeting the immune checkpoint using checkpoint inhibitors (CKI) can lead to long-term clinical response and cancer cure. Since the discovery of CTLA-4, several ICPs have been discovered, including programmed death-1 (PD-1), T-cell immunoglobulin domain and mucin domain-containing molecule-3 (TIM-3), T-cell immunoglobulin and ITIM domain (TIGIT), B and T cell lymphocyte attenuator (BTLA), lymphocyte activation gene (LAG3), V-domain Ig suppressor of T cell activation (VISTA), and Cluster of Differentiation 200 (CD200) (Fig. 2B) [40].

Interaction of PD1 (expressed on effector T cells) with PD-L1 (expressed by tumor cells and TME-associated myeloid cells) acts as an inhibitory signal and causes effector T cell exhaustion. While CTLA-4 is upregulated in activated T cells and competes with the co-stimulatory CD80/86 expressed on APCs, thereby negatively affecting T cell activation and function. While PD-1 and CTLA-4 are the most studied ICPs, other ICPs may be effective. Tumor cell-expressed ligands (CD155, CD112) binds to TIGIT and impact T cell- and NK-cell-mediated tumor recognition. TIM3 and LAG3 inactivate T cell function and induce exhaustion (Fig. 2B) [15, 40]. A schematic showing immune interaction between T cell and APC; T cell with tumor cells is shown in Fig. 2B. Loss of CD4 + T cells and an increase in the expression of inhibitory receptors such as CD160, CD244, CTLA4, LAG-3, PD1, TIGIT, and TIM3, leads to a rapid decline in T cell effector activity. Advanced technologies have provided a comprehensive understanding of the complexity of the tumor-immune interactions. By parsing the distinct type of tumor-specific TIME, immunotherapeutic responsiveness may be predicted, and novel therapeutic targets can be identified for developing successful therapies.

CKIs as a therapy for advanced lung cancer have lately gained traction. U.S. Food and Drug Administration (FDA) in 2015 approved Nivolumab (blocks PD-1) for the treatment of LUSC (and subsequently for all NSCLC histological types) after the first-line treatment with platinum doublet chemotherapy had failed. Under normal conditions, the immune checkpoint receptor programmed cell death-1 (PD-1) is expressed on activated T cells. PD-1 inhibits immunological responses from being overstimulated, while its ligand, PD-L1, is expressed on immune cells and tumor cells. The PD-1/PD-L1 pathway interaction contributes significantly to tumor immune evasion. The anti-tumor immunity mediated by T cells is resurrected by inhibiting their connection, offering a survival advantage in various advanced, resistant cancers. For patients with PD-L1 positive cancers who had progressed after chemotherapy, Pembrolizumab was approved in 2015. In October 2016, the FDA authorized pembrolizumab as first-line therapy for patients with high (≥ 50%) PD-L1 expression. Atezolizumab was approved for use in patients with advanced NSCLC who had progressed after chemotherapy. Research on the role of the immune system in the treatment and prevention of cancer has been substantial. Immunotherapy is now a trendy topic, thanks to a flurry of FDA approvals. Immunotherapy encompasses cancer vaccines, MAbs, and adoptive cell transfer in addition to checkpoint inhibitors and will be discussed in further detail in the subsequent sections.

### Tumor-specific vaccines

**Fig. 3:**
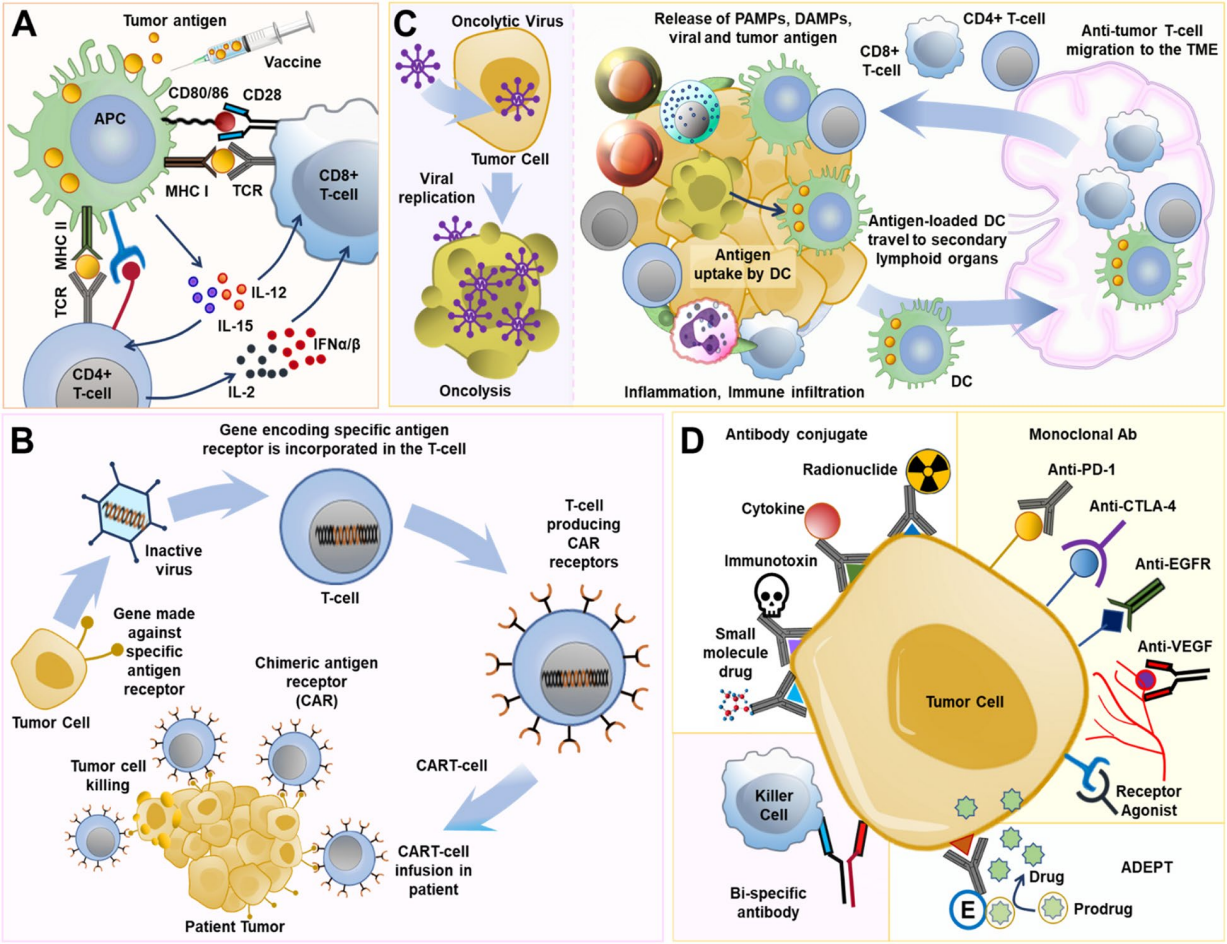
Different aspects of lung cancer immunotherapy. **A**: Lung cancer immunotherapy by using a tumor-specific vaccine to combat cancer. **B**: Donor or patient T cells are collected in vitro, followed by the introduction of Chimeric Antigen Receptor (CAR) receptors and mass-produced in the lab to combat cancer. Following infusion back to the patient, the CAR T-cells attack the patient's tumor. **C**: Oncolytic virus and lung cancer cell oncolysis. **D**: Monoclonal antibodies (mAbs) may be effective against lung cancer by targeting a specific section of the cancer cell

For long, vaccines have been old arsenals in medicine, primarily used to prevent the onset and spread of infectious disease, and to a smaller degree have been applied in oncology. The vaccines aim to promote antigen-specific immune responses in a patient by presenting TAAs to the individual’s immune system in the cancer environment [44]. Vaccine therapy aims to initiate or amplify adaptive anti-tumor immune responses by introducing tumor antigens to stimulate the host immune system to generate tumor antigen-specific effector and memory T-cell-based responses and not target non-malignant cells [45–47]. Vaccines targeting NSCLC have been investigated in several phase III trials throughout the last decade. Although they had a favorable toxicity profile and tolerability, almost all of them could not demonstrate survival advantages despite encouraging results in the preliminary phase II randomized trials. Tumor vaccination faces multiple challenges, and addressing them can lead to the path of therapeutic translation. Cancer vaccines suffer from limited penetrability in the tumor, wayning of the immuneresponse over time, and resistance. Multi-target vaccines generated against immunogenicity-optimized epitopes may address some of these challenges. Therefore, a greater knowledge of immune evasion mechanisms, designing effective formulations, and combination immunotherapy approaches (targeting TME and tumor cell-derived factors) can promote the development of the subsequent generation of cancer vaccines. The currently investigated vaccines, classified broadly into antigen-specific vaccines (peptide /protein vaccines, DNA vaccines, and vector-based vaccines) or whole-cell vaccines (allogeneic vaccines and autologous dendritic cell vaccines), are discussed in brief below (Fig. 3A).

#### Peptide/protein vaccines

The few protein-specific vaccines used in NSCLC are the CIMAvax epidermal growth factor (CIMAvax-EGF) vaccine, MAGE-A3, NY-ESO-1 and the BLP25 liposome vaccine (anti-MUC1). The CIMAvax-EGF, developed in Cuba, is a chemical conjugation of EGF with the P64 protein obtained from Meningitis B bacteria and the incomplete Freund's adjuvant Montanide ISA 51 [48]. The vaccine induces immune responses specifically against EGF, a molecular driver of cancer cells, aiming to block their proliferation. Its use is currently approved in the countries like Cuba, Peru, and Venezuela for treating stage IIIB and IV NSCLC patients who have progressed beyond the first line of chemotherapy. The CIMAvax-EGF was shown to be safe and immunogenic in patients with advanced NSCLC in a phase II randomized controlled study including 80 stage IIIB/IV NSCLC patients who had received a first-line chemotherapy [49]. Promising anti-EGF antibody response was documented in 51.3% of the vaccinated patients, and they survived significantly longer (11.7 months MS) than those that showed poor antibody response (3.6 months MS). Adverse events were recorded in fewer than a quarter of grades 1 and 2 patients. Subsequently, a phase III study was published in August 2016, showing the results of OS, safety, immunogenicity, and serum EGF concentration of 405 stage IIIB/IV NSCLC patients post-CIMAvax-EGF vaccination [50]. After completion of the first line of chemotherapy, patients were randomly assigned at a ratio of 2:1 for the vaccine with best supportive care (BSC) or to the control group. The survival was statistically higher (HR, 0.77; *P* = 0.036) in the treatment arm with an MS of 12.4 months for the vaccinated group contrasted with 9.4 months for the control patients. In January 2017, a new randomized phase I/II clinical trial against NSCLC with CIMAvax-EGF combined with the MAB Nivolumab (NCT02955290) began, for which the results are awaited.

Another type of protein targeting tumor vaccines is the ones that targets the cancer testis antigens (CTA) that include the New York oesophageal squamous cell cancer (NY-ESO-1) and the melanoma-associated antigen-A3 (MAGE-A3) antigens in case of NSCLC. The normal expression of CTA is primarily found in the male germ cells in the testis and rarely in the female ovary and trophoblast, while in some cases, due to genetic mutations, they become upregulated in a proportion of different malignant tumor types [51]. The MAGE was the first CTA to be identified, and its expression is evident in almost 30%—50% of NSCLC patients, especially in LUSC incidences [52, 53]. The phase III MAGRIT study evaluated the safety of a recombinant adjuvanted MAGE-A3 in patients with resected MAGE-A3 positive NSCLC. The trial included 2,312 patients with resected stage IB, IIA, or IIIA NSCLC. The vaccine comprises a recombinant protein comprising the MAGE-A3 and the fusion protein D of *Haemophilus influenzae,* along with the vaccine adjuvant AS02B. The trial's primary goal was to investigate disease-free survival (DFS). However, the study's final results did not show an improved DFS in the MAGE-A3 treated compared to the placebo control group (60.5 *vs.* 57.9 months, respectively) [54].

Similarly, NY-ESO-1, another CTA candidate, is expressed approximately in 30% of lung cancer specimens [55]. Many of its beneficial roles include a prognostic and a predictive factor for adjuvant and neoadjuvant chemotherapy treatment efficacy in NSCLC, and the capacity to induce specific antibodies in serum along with activation of the helper CD4 + and cytotoxic CD8 + T cells have already been demonstrated [56, 57]. Two concurrent phase I trials are at present recruiting patients to assess the safety and the immune response of ID-LV305 (immunotherapy targeting DCs in individuals with advanced cancer with the tumor cells expressing the NY-ESO-1 protein, NCT02122861) and of IDC-G305 (a new vaccine candidate containing recombinant NY-ESO-1 antigen and GLA-SE as an adjuvant, NCT02015416) in patients with NSCLC along with few other types of cancer patients. Another antigen expressed on NSCLC tumors is the mucin 1 (MUC-1) glycoprotein, which stimulates tumor cell proliferation pathologically via its cell surface receptor interaction [58]. It was chosen as a target for the development of the synthetic lipopeptide-based vaccine Tecemotide (L-BLP25), which was proved to be immunogenic and well-tolerated in a phase I study and was demonstrated as maintenance therapy for stage IIB-IV NSCLC patients through the achievement of stable disease or objective response, reported after the first-line chemotherapy in another phase IIB trial. In the phase III START trial, the treatment group showed no change in OS compared to the placebo-controlled groups (MS was 25.6 months vs. 22.3 months) [59–61]. Later, several studies, including the phase III START2 and INSPIRE trials were undertaken, but they were terminated owing to negative findings from the phase I/II Japanese EMR 63,325–009 study in unresectable stage III NSCLC patients. Use of pattern recognition receptor (PRR) activators and supramolecular peptide conjugates may enhance the potency of peptide vaccines. Hence further research is necessary to enhance the efficacy of peptide vaccines. Currently, personalized peptide-based vaccinations are being investigated for efficient therapeutic output.

#### DNA vaccines

DNA vaccines involve the insertion of a plasmid containing a particular DNA sequence encoding the target antigen to elicit specific immune responses in the presence of the antigen in situ. This approach is cost-effective and can be repeatedly administered. Another advantage is that the antigen post-expression can be presented by MHC class I and II, triggering CD4 and CD8 T cells and humoral immunity. While cytosolic sensors can recognize double-stranded plasmid DNA, which stimulates the innate immune response. Using a genetically engineered bi-transgenic Kras^G12D^ inducible mouse (TetO-Kras4bG12D/Scgb1a1-rtTA) lung cancer model, Weng et al. used the Kras DNA vaccination. Vaccination yielded an efficient antitumor response and effectively targeted Kras-driven lung tumors [62]. MAGE-A3 protein (recMAGE-A3) vaccination has been used to target MAGE-A3, expressed in melanoma and NSCLC. Though effective in mouse melanoma models, when administered with or without adjuvant as a part of the large randomized MAGRIT MAGE-A3-positive NSCLC phase III trial demonstrated no advantage over the placebo [63]. DNA vaccines work in animal models but did not show promising results in clinical studies, necessitating the exploration of novel strategies. A comparison of xenogeneic antigens, neoantigens, and TAA in relation to therapeutic efficacy needs to be determined. Future research needs to investigate combination therapy approaches potentiate (targeted at activating antigen response and immunosuppression) to mediate synergistic and sustained immunogenic response in lung cancer. Another key area to investigate is the application of DNA vaccines in oncogenic virus-induced/activated cancers, including lung cancer. Advances in ex vivo DCs pulsing, nanotechnology, and surface functionalization approaches can help increase the efficacy of DNA vaccines. Considerations of immunodominance versus tolerance of immunogenic epitopes, poly-specific and poly-functional DNA vaccine, combination TAAs and neoantigens in a vaccine may boost vaccination-associated immunogenicity [64].

#### Vector vaccines

Vector-based vaccines are the constructs developed by manipulating specialized bacteria, viruses, yeast, or other structures to express any recombinant antigen. The TG4010 is a viral vector vaccine comprising a modified Vaccinia Virus Ankara (MVA) that encodes the human MUC1 and interleukin 2 [65]. Rochlitz et al*.* 2003, reported a good safety profile in a phase I clinical trial, where 13 patients having different solid tumors including lung cancer, were subjected to increasing doses of TG4010. Among them, one lung cancer patient showed a considerable reduction in the extent of metastasis over 14 months [66]. A phase II randomized clinical trial consisting of stages IIIB and IV NSCLC patients potentially pointed towards using TG4010 in combination with chemotherapy in first-line advanced or metastatic NSCLC for better chemotherapy results. [67]. The patients were administered the TG4010 in combination with the first-line chemotherapy (Cisplatin plus Vinorelbine doublet) or received the vaccine alone, and 29.5% of the patients who received treatment in the combination arm had a radiological response. A phase II trial (NCT00793208) that combines TG4010 with Nivolumab is ongoing [68]. Apart from this, other adenovirus vaccines expressing melanoma-associated antigen 3 (MAGE-A3) and MG1 maraba oncolytic virus (MG1-MAGEA3) were tested in phase I/II dose-escalation trial (NCT02879760) testing the combinatorial efficacy of the vaccine with Pembrolizumab, is presently recruiting NSCLC patients who have shown signs of radiological progression with at least one cycle of platinum-doublet chemotherapy [69]. The MAGE-A3, alone and in combination with MG1-MAGEA3 was tested in solid tumors, including lung cancer (NCT02285816) [69]. A better understanding of the molecular mechanism may enhance the efficacy of vector-based vaccines.

#### Dendritic cell vaccines

Cell-based immunotherapy helps immune cells identify tumor antigens and target cancer cells. This potential therapeutic immunotherapy technique is mainly explored in the context of dendritic cell-based vaccines, as DC therapy is safe and can elicit robust antigen-specific T cell responses owing to their antigen-presenting abilities [44]. Since the FDA authorization of Sipuleucel-T in April 2010 to treat metastatic prostate cancer, DC vaccines have progressed significantly, and several clinical trials are ongoing. A promising approach is the intra-tumoral delivery of autologous DC vaccine (CCL21 gene-modified DCs or AdCCL21-DC) targeting lung cancer. Lee et al. reported significant activation of CD8 + T cell tumor infiltration and antigen-specific immune response while using AdCCL21-DC in phase I clinical trial on stage IIIB, stage IV, or recurrent NSCLC (NCT00601094) [70]. Following the exciting results, another follow-up phase I trial is underway to evaluate the efficiency of pembrolizumab and AdCCL21-DC in combination on advanced-stage NSCLC patients (NCT03546361). The mechanistic effect of intratumoral CCL21-DC combined with anti-PD-1 therapy was further evaluated on murine NSCLC models [71]. Abascal et al. recently used murine CD103 + cDC1 (conventional DC type I) cells to produce soluble FLT3L (FLT3L cDC1) and conducted in situ vaccination experiments on anti-PD1 resistant murine NSCLC models and reported enhanced anti-tumor efficacy compared to non-modified cDC1 cells. Emerging research suggests DC vaccination may increase patient survival, calling for developing next-generation DC vaccines and testing new DC vaccine-immunotherapy combinations [71]. Nevertheless, the unique biology and classification of DCs, immune tolerance, weak and limited lifespan hamper their persistent and effective cancer immunity, and the production process are challenges that need to be addressed [72]. The role of different types of DCs (Mo-DC, cDC1, cDC2, pDC) and DC-derived exosomes in the context of the DC vaccine development may be further evaluated.

#### Allogeneic vaccines

Allogeneic vaccines contain non-self-cancer cells as the antigen source. Cancer cells of one patient are harvested and administered in another patient with the same tumor type, post necessary modifications and processing [73]. One such vaccine is the Belagenpumatucel-L. It is prepared by transfecting four radiated allogeneic NSCLC cell lines (H460, RH2, SKLU-1, H520, of which 2 are LUAD, one LUSC, and one LCC cell line) with a plasmid bearing the antisense of transforming growth factor β2 (TGF-β2) [74]. High levels of TGF-β have been correlated to immune suppression and worsening prognosis in NSCLC patients [75]. Inclusion of the antisense transgene in this vaccine inhibits TGF-β2 intending to increase immunogenicity. To assess its efficacy, a phase III randomized controlled trial (STOP), with 532 stage III/IV NSCLC patients who had no disease progression after a first line of platinum-based chemotherapy, was conducted that compared Belagenpumatucel-L with placebo. However, the study did not satisfy the primary endpoint as no difference in MS was observed between the vaccinated and the placebo arms (20.3 months vs. 17.8 months respectively, HR 0.94, *P* = 0.594). Similarly, another vaccine candidate comprising of autologous or allogeneic NSCLC cells plus GM.CD40L expressing K562 cells, when studied through phase I and II trials, could yield no affirmative results in terms of MS in NSCLC patients. Currently, two other allogeneic vaccines the Tergenpumatucel-L (NCT02460367, with 16 participants in a phase Ib/2 trial) and Viagenpumatucel-L (NCT02439450, with 121 participants in a phase Ib/2 DURGA trial), are being investigated in combination with ICIs. The major challenges of monotherapy include intratumoral heterogeneity, allogeneic vaccine-induced mutational divergence, and tumor escape, vaccines developed from cell lines/cellular components (non-self) may not reflect actual tumor antigens and tumor/ TME-induced immunosuppression. Hence better transcriptome analysis for antigen selection, including neoantigens, tackling immunosuppression, and combining T cell-based immunotherapies may become clinically translatable. Various clinical studies are underway on various solid tumors, and any success strategies may be expanded to lung cancer therapy.

### Adoptive cell therapy

Adoptive cell therapy (ACT) utilizes tumor-reactive immune cells from patients, especially different types of T cells, that are grown and genetically engineered ex vivo before being re-administered to the patient as a therapy to identify and target cancer cells. In this regard, the most commonly used are Chimeric antigen receptor (CAR)-modified T cells (CAR T) therapy, Tumor-infiltrating lymphocyte (TIL) therapy, engineered T-cell receptor (TCR)-therapy, and Natural killer (NK) cell therapy [76]. Among these, the CAR-T cells and TCRs are both genetically modified synthetic biology approaches to target particular tumor antigens and exhibit prominent therapeutic effects [77, 78] (Fig. 3B). CAR-T cell immunotherapy has an 80–90% remission rate in hematological malignancies, and FDA has authorized CD19-targeting CAR-T for treating hematological cancers. This recent success with CAR-T therapy has changed the landscape of cancer therapy and spurred research efforts to translate these curative benefits to solid tumors like lung cancer [79].

### CAR T cell therapy and lung cancer targeting

**Fig. 4:**
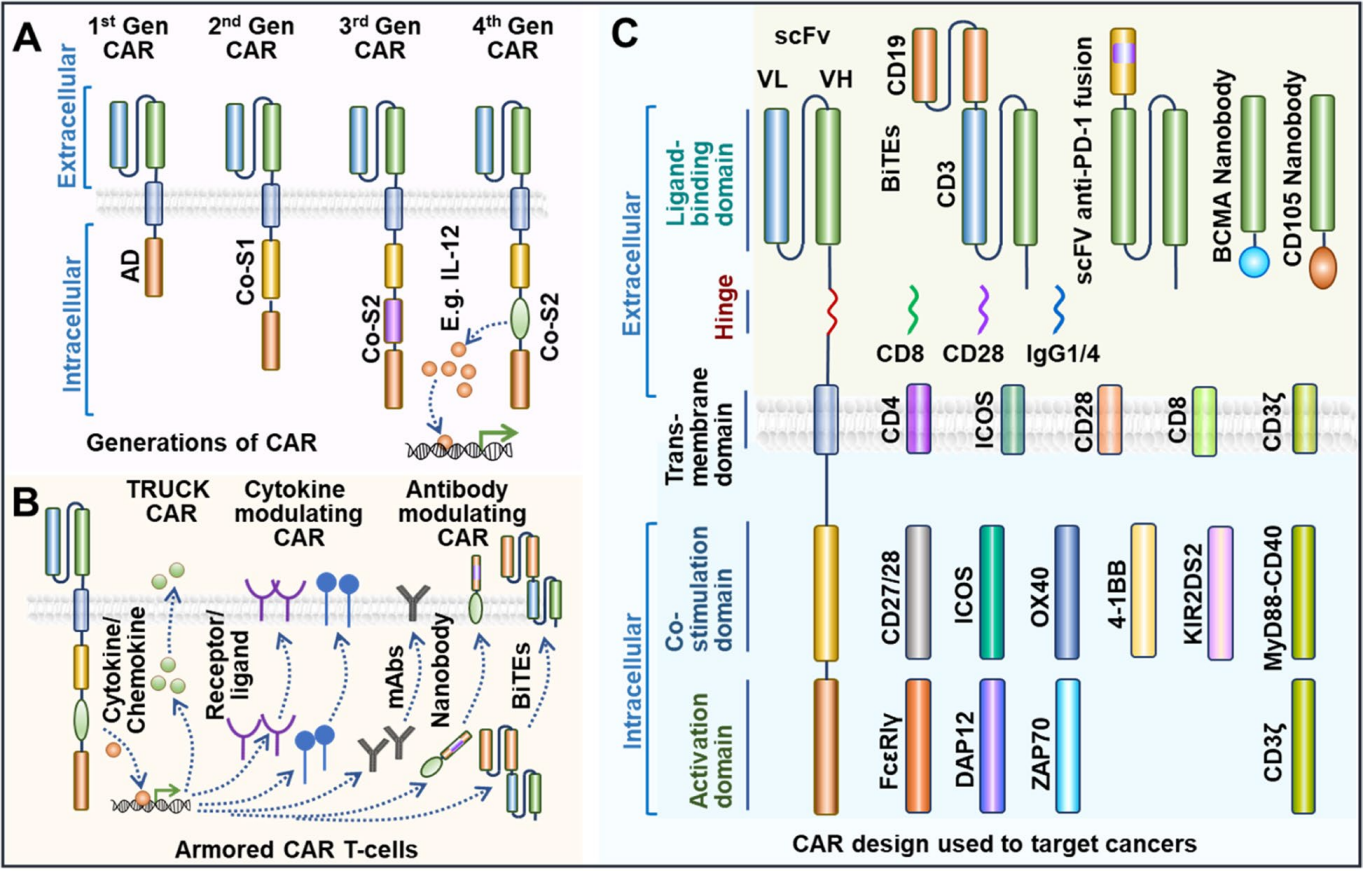
Schematic representation of CAR architectural design. **A**: Evolution of CAR design through generations. **B**. Armored CAR T-cell design and functional mechanism. **C**. Schematic enumerates various building components that correlate to different CAR segments that may be exploited as CAR construction components. Gen: Generation; scFv: single-chain variable fragment; AD: activation domain; Co-S1: Co-stimulatory domain 1; Co-S2: Co-stimulatory domain 2; VL: variable light; VH: variable heavy; TRUCK: T-cells Redirected towards Universal Cytokine Killing. BiTEs: Bispecific T-cell engager

CAR-T cells are created genetically engineering autologous or allogeneic T cells in vitro by modifying T-cell receptors to identify and bind to antigens on cancer cells. Chimeric antigen receptor (CAR) are synthetic designed receptors, retrovirally transduced into T cells, and consists of three domains (Fig. 2, 3). CAR primarily consists of an extracellular antigen recognition domain (ectodomain), a transmembrane domain, and an intracellular signal transduction domain (endodomain). The ectodomain that determines the CAR’s affinity comprises an antibody-derived single-chain variable fragment (scFv) composed of the antigen-binding zone, including both the heavy and light chains of a monoclonal antibody (Fig. 4) [80]. On the one hand, the transmembrane domains connect the ectodomain via a hinge; on the other hand connect the endodomain, thereby anchoring the CAR to the cell.

membrane. The CAR's intracellular domain has an activation domain (AD) and one or two co-stimulatory domains (Co-S1, 2) and can modulate the length, flexibility, surface density, downstream signal, and aggregation potential of CAR and thereby CAR-T functions (Fig. 4) [80, 81]. The activation domain is often associated with costimulatory molecules, which activate T cell activity and contribute to T cell proliferation and longevity. Phosphorylation of the immunoreceptor tyrosine-based activation motifs (ITAMs) of CAR endodomains activate and costimulate T lymphocytes. CAR structure evolution has seen several generations with hierarchical assimilation of functional modules (Fig. 4A) [82]. While the first-generation CAR lacked a costimulatory domain, the second and third-generation CAR adapted one and two costimulatory domains, respectively. Fourth-generation CAR-Ts combine the direct tumoricidal activity of CAR-Ts and the ability to overcome the immune-modulating ability of the tumor microenvironment without the systemic side effects (Fig. 4A).

CAR-T cells of this generation, called armored CAR T-cells, express proteins to minimize immunosuppression and increase anti-tumor activity (Fig. 4B) [83]. These CAR modifications can be summarized as follows, T-cells Redirected towards Universal Cytokine Killing) TRUCK, cytokine modulating CAR, and antibody modulating CAR. TRUCK CAR secretes cytokines to interfere with the immunosuppressive properties of solid tumors. By putting brakes on the immune-suppressive cytokine environment, it may be possible to enhance CAR T-cell and resident immune cell antitumor potential [80]. Cytokine modulation CARs depend on engineering CARs to express specific receptors, and ligands can help regulate CAR T-cells reaction to cytokines and may also affect cytokine function (Fig. 4B). To specifically target cancer antigens, CARs can be engineered to produce antibody-like proteins, called antibody modulating CARs. Another popular CAR design aspect is a nanobody or VHH antibody, composed of a single antibody's variable heavy chain or heavy chain-only antibodies (HcAbs) [84]. These armored CAR T-cells may boost solid tumor targeting efficacy due to their high affinity towards antigen, compactness, optimal stability, and manufacturability [85]. Bispecific T-cell engagers (BiTEs) are examples of innovative CAR design, where adaptive therapeutic benefit is achieved by a conjunction of two scFvs with differing antigenic specificities [86]. BiTEs are designed to bind to proteins on T cells and proteins on tumor cells, bringing them spatially closer, establishing an immune synapse, and unleashing immune cell killing (Fig. 4). Figure 4C lists several components that may be utilized as construction blocks corresponding to various CAR segments. CAR T treatment confronts various challenges when targeting solid tumors. However, increased attempts are being undertaken to target lung cancer in light of developments in synthetic biology, creative CAR design, and effectiveness against blood cancer [82].

Carcinoembryonic antigen (CEA) is strongly expressed in lung cancer compared to healthy cells, and high CEA expression is related to poor prognosis and metastasis. Therefore, anti-CEA CAR-T cell therapy and its safety efficacy are evaluated on CEA-positive lung cancer patients (NCT02349724, NCT04348643). Like CEA, Mucin 1 (MUC1) is also highly expressed in lung cancer, promotes metastasis, and is thus an exciting target for ACT against lung cancer [87, 88]. CAR-T cells targeting MUC1 successfully eliminate NSCLC tumor cells [88] and are evaluated in clinical trials for lung cancer (NCT03525782, NCT02587689, and NCT05239143). A third-generation anti-PSCA/MUC1/TGFβ/HER2/Mesothelin/Lewis-Y/GPC3/AXL/EGFR/B7-H3/Claudin18.2-CAR-T has been evaluated in an interventional Phase I trial (NCT03198052). Another interesting target is the cluster of differentiation 276 (CD276). CD276 is a new cancer diagnostic marker and an indicator of immunological status and prognosis that correlates favorably with the NSCLC tumor stage [89]. The silencing of CD276 modulates integrin signaling to ameliorate lung cancer metastatic potential [90]. Anti-PD-1 and anti-PD-L1 antibodies have radically improved NSCLC therapy with significantly increased ORR and PFS but suffers from multiple challenges. Recently PD-L1-targeting CAR T cells have been found effective in xenograft NSCLC tumors with high or low PD-L1 expression [91, 92]. MSLN-CAR T cells secreting PD-1 nanobody is being explored in an interventional phase I trial for NSCLC (NCT04489862). Immune infiltration in a tumor is a significant hurdle in effective ACT therapy. NSCLC tumors produce substantial amounts of the chemokine CXCL13, and an intelligent design would be to express its single receptor CXCR5 on the CAR T cells for enhanced CAR T cell tumor infiltration and heightened efficiency. An exploratory study of anti-EGFR CAR T cells modified by CXCR 5 is under phase I trials in NSCLC (NCT05060796) [93]. A third/fourth generation GPC3-CAR-T cell was engineered to target Glypican-3, a cell membrane proteoglycan and a potential marker specifically for LUSC and also for LUAD [94]. The GPC3-CAR was also engineered to express TGFβ targeting CAR (GPC3/TGF-CART), is effective in in vitro and in vivo experiments, and is under a phase I interventional investigation for LUSC (NCT03198546). TGFβ-CAR T cells can reduce the immunosuppressive tumor microenvironment making it more conducive for T cell activation [95]. A recent study examined PD-L1-targeting CAR T cells in PD-L1 high and low xenograft NSCLC tumors. PD-L1-CAR T cells exhibited robust effector T cell function and destroyed PD-L1 high and PD-L1 low tumors. Local irradiation improved PD-L1-CAR T cell efficacy and can be an innovative and effective strategy against PD-L1 low NSCLC patients [96]. Some exciting target antigens used for generating CAR against lung cancer include MUC-1, CEA, MSLN, HER2, GPC3, ROR1, and EGFR [97]. Although lung cancer CAR T immunotherapy is in its infancy, several challenges must be overcome before it can usher in widespread clinical implementation [98].

Developing an ACT for targeting lung cancer presents a big challenge. However, selecting the ideal TSA or TAA with minimal expression in normal tissue, eliminating off-tumor adverse effects and the immune-tolerant state posed by the tumor microenvironment, needs to be considered before developing CAR-T cells and TCRs for targeting NSCLC. Tackling CAR T -associated toxicity, antigen escape, heterogeneity of antigen, reduced CAR T proliferation in the tumor microenvironment, and CAR T tumor infiltration needs innovative synthetic biology approaches. Integrating appropriate modules to sense specific intra- and extracellular signals and actuator modules to coordinate precise transcriptional or translational control will effectively address the current lacunae. The use of multiple CARs on the same or different cell types (e.g., CD4/ CD8/ NK cells) and the ability to spatiotemporally controllable transient CAR activation using switchable CARs (inducible by ultrasound/ light/ drug/ adaptor) can strengthen the development of effective CAR T therapy [99]. Also, generating CAR-T cells with safety switches with inducible caspase-9 gene may be a successful approach [100–102]. To improve CAR T safety, novel self-driving and self-destruct CAR architecture are being engineered. CAR design, including multiple antigen-targeting abilities, can effectively address heterogeneous antigens in lung tumors [78, 98]. Another approach is to generate personalized.

CAR T for specific lung cancer genotypes. The rapid use of artificial intelligence (AI) in data analysis and synthetic biology design using CRISPR [103] may help scientists design synthetic receptors in order to correlate various chemical recognition events (e.g., SynNotch) [104]. Some researchers are trying to mass-produce CAR-T “off-the-shelf” cells, and this approach might make their CAR T therapy easier and cheaper. There is a need to identify neoantigens and develop enhanced high throughput screening tools to ensure that all structural components of the CAR T cells are optimized to target lung cancer.

#### T-cell receptor (TCR) engineering and lung cancer

**Fig. 5:**
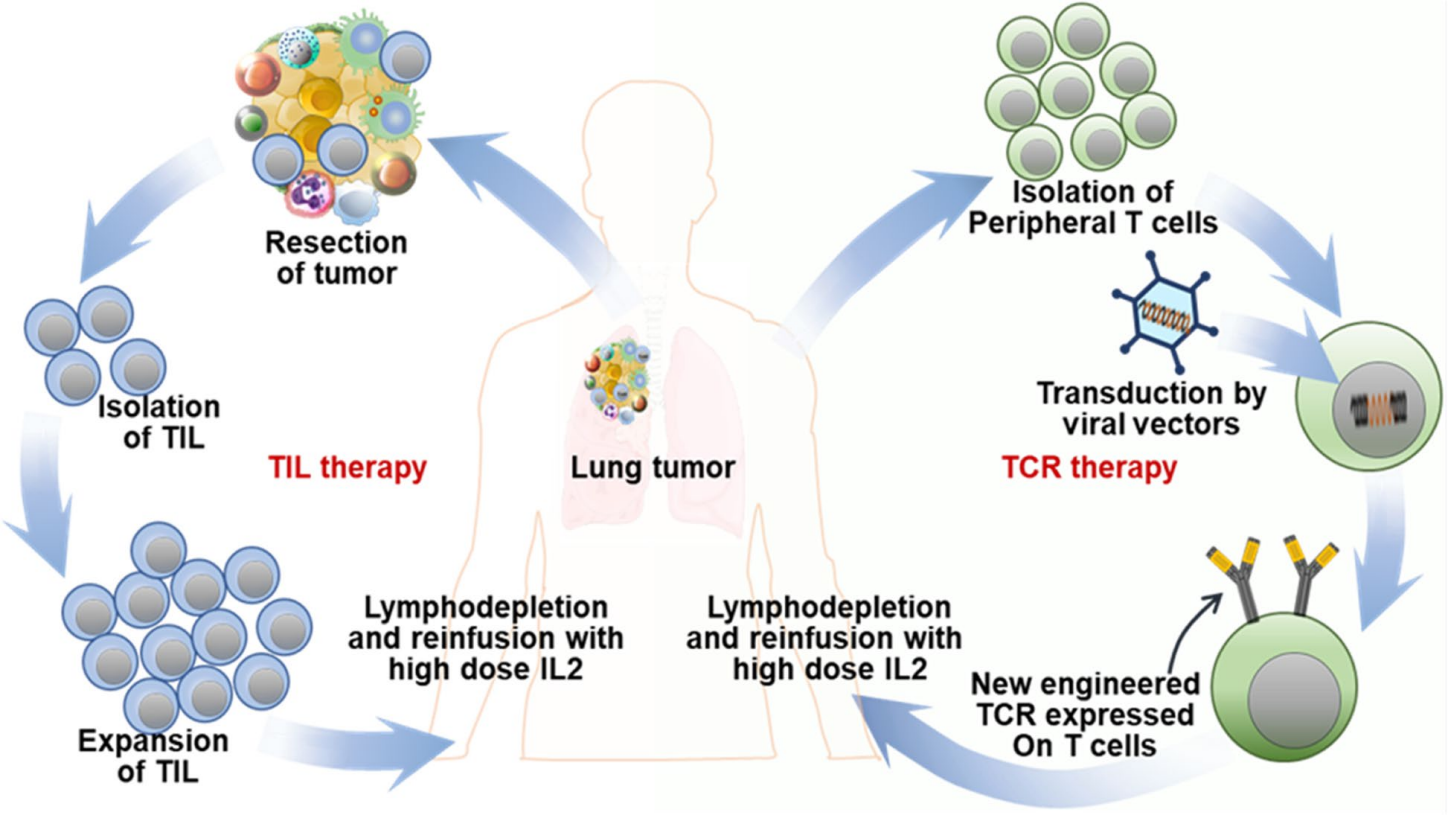
Schematic showing TIL and TCR T cell therapy. TCR T and TILs are isolated from the patient and multiplied in the laboratory before being reintroduced to the patient

TCR immunotherapy employs the innate mechanism of T cells to target tumor antigens by genetically modifying T cells ex vivo to express cancer-antigen-specific T cell receptors (TCRs) generated via TCR-engineering of patient-isolated T cells (TCR T) (Fig. 5) [105]. TCR therapy has distinct advantages over CAR T therapy targeting solid tumors like lung cancer [77]. TCRs identify only specific oncogenic peptides presented by human leukocyte antigen (HLA) class I on the surface of a tumor cell or an APC [106]. TCR T lymphocytes may target tumor mutation-derived neoantigens in a highly selective and non-toxic way, and a majority of clinical trials are focused on solid tumors.

Multiple in vitro and in vivo investigations have shown the anti-cancer efficacy of TCR-T cells engineered to target lung cancer-specific antigens. Recently autologous TCR T-cell therapy against a lung tumor-specific protein called NY-ESO-1 is gaining traction. Letetresgene autoleucel (GSK3377794) is a first generation of TCR T-cells designed to target NY-ESO-1 and showed an objective response in Multiple Myeloma and myxoid/round cell liposarcoma trial (NCT03168438, NCT02992743). The safety and efficacy of Letetresgene autoleucel were evaluated after infusing back anti-NY-ESO-1 TCR to patients following lymphodepleting chemotherapy in a phase I trial that is now completed (NCT02588612). Other phase I trials using anti-NY-ESO-1 TCR transduced T cells alone or in combination with pembrolizumab on advanced NSCLC patient is underway (NCT03029273, NCT03709706). Another exciting target for TCR-T cell therapy is Kita-kyushu lung cancer antigen 1 (KK-LC-1), reported to be higher in LUAD than LUSC and exhibited a higher association with higher TMB tumors [107]. A clinical trial is currently investigating the safety and dosing of TCR Gene therapy targeting KK-LC-1 in KK-LC-1 + lung cancer patients (NCT05035407). TCR-T therapy has made a breakthrough in many tumors; however, there are few safe and effective targets due to possible off-target, dose-limiting cytotoxicity, autoimmune toxicity, and cytokine-related toxicity. Long-term safety evaluation studies with TCR-T cell therapy are also underway for lung and other cancers (NCT05292859, NCT05194735) and understanding their efficacy concerning somatic mutation and HLA typing (NCT05124743). A recent study used autologous TCR T therapy to target the hot spot KRAS G12D mutation in pancreatic cancer [108], with significant tumor regression and an overall partial response of 72%. Despite the survival of TCR-transduced T cells in the circulation, a second patient with the identical KRAS mutation and HLA allele did not benefit from T cell infusion, suggesting other potential TCR T failure mechanisms. The KRAS genetic variants are significant in lung cancer, especially in LUAD; the G12D mutation corresponds to ~ 3% of patients [109], and therefore a similar targeted TCR T therapy can bring a sea change in lung cancer treatment.

Though TCR T cell therapy is compelling, several challenges must be addressed for widespread clinical use [105]. Deficiencies in the antigen-processing and presentation machinery, downregulation of tumor HLA molecules and target mutations, augmentation of immune-suppressive cytokines, and complimentary activation of pathways (e.g., Wnt) inducing T cell tumor exclusion by the tumor/ TME can lead to attenuation of the TCR T therapy response [110, 111]. Moreover, HLA-coding genes are highly variable in the human genome, with about 20,000 alleles complicating TCR T HLA restriction. Unlike CAR T therapy, TCR T therapy TCRs are restricted to commonly found HLA alleles, such as HLA-A*02:01. TCR specificity is encoded by two distinct gene regions (TCR α and β transcripts) [112]. While doing TCR sequencing, intermixing can create ambiguity over which TCR α sequence corresponds with which TCR β sequence. Therefore, clonal T-cell isolation methods like limiting dilution, single Cell RT-PCR, and Single-Cell RNA Sequencing (scRNAseq) can be used [113]. Mispair heterodimerization of the TCR constant region of α and β chain of endogenous and engineered TCRs may lead to non-productive TCRs, unexpected autoantigen specificity, competition with engineered TCR, and may cause unwanted graft-versus-host-disease (GVHD). Techniques like murinization (integrating murine-derived TCR), the introduction of an additional disulfide bond (at residue 48 of Cα and residue 57 of Cβ), introducing stabilizing mutations (α-LVL) in α chain, domain swapping (Cα glycine and Cβ arginine), single-chain TCR, and combinations can improve TCR efficacy and safety profile [105]. Another issue is choosing and pre-clinically testing amongst the multiple antigen-specific TCR sequences discovered. An exciting tool is HLA restriction, where individually cloned HLA in COS7 cells are cocultured with TCR T cells to detect T cell activation. TCR affinity/avidity studies evaluate the association rate, dissociation rate, and binding constant using surface plasmon resonance. Cotransfected CD4 + T cells augment antitumor effects by boosting CD8 + T cell proliferation and survival [114]. Therefore, more studies with potential synergy with other immunotherapy, radiotherapy, and chemotherapy must be evaluated [106]. Novel genome engineering techniques, advances in single-cell genomics, and enhanced know-how through TCR T therapy trials may help design more effective TCR T cell therapy in lung cancer.

#### Tumor-infiltrating lymphocyte (TIL) and lung cancer

TILs can naturally recognize and target tumor cells, but the tumor cells usually evade this TIL-based immune response. TILs are often enriched with tumor-antigen specific T cell clones compared to T cells in peripheral blood and hence used for TIL therapy [115]. This novel immunotherapy approach uses TILs isolated from the patient's tumor and expanded ex vivo using recombinant IL-2 (rIL-2). IL-2 promotes TIL proliferation, activation, and tumor-killing activity [116]. Prior to TIL therapy, the patient is subjected to non-myeloablative lymphodepletion to eradicate the immune-suppressive TME. Billions of these TILs are then reintroduced in the patient, where they can proliferate, recognize tumor cells, and effectively ablate them (Fig. 5). TILs are primarily studied in solid tumors, including lung cancer [115].

In a similar context, ACT with TILs has been evaluated in NSCLC patients. One of the earlier studies examined the usefulness of TILs as a post-operative therapy for stage II–III NSCLC patients where tissue samples were surgically resected from the primary lung lesions of NSCLC patients [117]. Isolated lymphocytes and cancer cells were grown in a medium supplemented with rIL-2, and TILs were infused in stage stratified patients and subcutaneous injections of IL-2 daily until the maximum tolerable dosage was reached. The TIL arm had a favorable MS compared to the standard of care treatment arm (22.4 vs. 14.1 months). Recently, according to the results of a small phase I trial, TIL therapy (≥ 20 × 10^9^ to 10^10^ CD3 + cells) along with IL-2 was found to be a feasible treatment option with a manageable toxicity profile [118]. It was observed that the TILs expanded in 95% of NSCLC patients with metastasis and had disease progression on Nivolumab (Opdivo). Another group investigated the effect of human double-negative T (DNT) cells (CD3 + CD4-CD8-) in targeting advanced lung cancer in vitro alone or in conjunction with Nivolumab (anti-PD-1 antibody). They observed that both patient- and healthy donor-derived DNT cells, expanded ex vivo, exhibited similar cytotoxicity against lung cancer cells [119]. It was also noted that DNT cells derived from healthy donors could considerably inhibit the growth of xenografts obtained from advanced-stage lung cancer patients, and the anti-cancer effect was further improved by the anti-PD-1 treatment that influenced augmented tumor infiltration of DNT cells. Autologous TIL therapy was also effective in a phase I trial on metastatic PD-1-resistant lung cancers (NCT03215810) [120]. However, the widespread application of TIL therapy has been mitigated by the demand for producing sufficient TILs in a stringent time period. Applications of rapid expansion protocols complying with good manufacturing practice conditions have improved the deficit [121]. Currently, a phase II study (NCT02133196, 85 participants) is recruiting patients to revise the utility of using autologous young TILs derived from NSCLC patients in combination with drugs like Aldesleukin, Fludarabine, and Cyclophosphamide. Further clinical trials are anticipated to predict the appositeness of TIL-based ACT in this new era of immunotherapy.

### Oncolytic viruses for lung cancer immunotherapy

Oncolytic Viruses (OVs) are genetically modified viruses that can identify, infect, and lyse diverse cell types in a tumor microenvironment, which can halt and often reduce tumor development [122]. They either possess a natural tropism to the cancer cells or can be genetically manipulated to recognize specific targets displayed by the cells. These targets often comprise of the nuclear transcription factors like human telomerase reverse transcriptase, osteocalcin, cyclooxygenase-2, prostate-specific antigen, or surface markers like folate receptor, prostate-specific membrane antigen, endothelial growth factor receptor, CD20, and HER2/Neu [123]. Moreover, it is well-known that different evasion mechanisms are in play in the tumor microenvironment that directly or indirectly downregulates the immune response, aiding the disease progression even in immunocompetent patients [124]. It has been observed that the OVs may stimulate the immune system against the tumor cells, hence affecting the establishment of an anticancer response [124–126]. Therefore, the clinical application of OVs emerges as a viable approach to induce an inflamed state in the tumor microenvironment whereby the immune system can detect and kill the abnormal cells [126, 127]. Additionally, the viruses exhibit a variety of pathways that direct the infected cells toward lysis, resulting in tumor cell death and enhancing immunotherapy effectiveness [128] (Fig.**3**C). Several such genetically modified OVs are currently undergoing investigation for lung cancer to determine their applicability and efficacy in the disease scenario. For example, the cytotoxic effect of oncolytic Herpes Simplex Virus-1 (HSV-1) regulated miRNA145 delivery was tested in vitro in human NSCLC cell lines (A549, H460, H838, and H197), showing therapeutic potential [129, 130]. Likewise, it has been proven that Coxsackievirus B3 (CVB3) holds precise oncolytic activities in nine human NSCLC cell lines. Also, it has been observed that intralesional injections of the virus in transplantable lung tumor models led to significant regression. The virus-infected NSCLC cells secreted ATP, abundantly expressed calreticulin on their surface, and translocated extranuclear HMGB-1, which are innate immune response markers that indicate immunogenic cell death (ICD) [131, 132]. Similarly, the application of oncolytic vaccinia viruses (OVVs) having three individual genetic backbones has been investigated in preclinical studies [133–135]. Moving onward, the applications of OVs are also under investigation through several completed and ongoing clinical trials. Lysogenic Adenovirus has an extensive tissue tropism, exploited in a two-intervention armed clinical trial (NCT01574729) including 58 patients, was conducted to evaluate an oncolytic Adenovirus (rAd-p53) mediated wild-type p53 gene transfer in stage III or IV NSCLC patients [136, 137]. 33% of patients in the experiment received a combination of rAd-p53 injection (through intratumoral or bronchial artery access) and chemotherapy instillation via the bronchial artery, while the rest (control group) received only the chemotherapy treatment. It was observed that the combinatorial treatment group showcased an extended disease progression than the control group (MS, 7.75 vs. 5.5 months; *P* = 0.018) of patients. Two patients with stage III NSCLC who received the combination treatment showed a complete response.

Similarly, much earlier in 2007, the potential of Seneca Valley Virus isolate 001 (SVV-001, now NTX-010) as an OV came to the forefront [138]. Additionally, it was reported that even the maximum viral dosage was well tolerated in SCLC and other malignancies, with predictable viral clearance kinetics and intra-tumoral viral replication [139]. However, data obtained from a recently published randomized placebo-controlled, double-blind, phase II clinical trial (NCT01017601) involving 50 patients with advanced-stage SCLC without advance of disease after platinum-based chemotherapy treatment suggests that the first-generation SVV-001 OV as a single agent may be incapable of generating desired clinical efficacy in the patients [140]. OV therapy faces multiple challenges, including ECM-based barrier to viral spread and tumor penetration leading to inadequate tumor trophism, passive targeting, previous immunization-associated anti-viral immune response, and tumor hypoxia inhibiting viral replication and functions. Different viral engineering approaches can help address some of the challenges, including using RGD-motifs, scFv fusion with capsid protein, bi-specific adaptors, capsid modification, stealthing, use of hypoxia-responsive promoters, novel theranostic modalities, and considering different serotypes. Other ongoing and completed clinical trials estimating the applicability of OVs in lung cancer are listed in Table 1.

**Table 1:** Completed and current clinical trials evaluating the applicability of Oncolytic Viruses in lung cancer

Clinical Trial ID	Oncolytic virus	Virus type	Transgene/ Target	Combination	Cancer	Status	Duration	Sponsor/ Agency
NCT 02,879,760	Ad-MAGEA3 with MG1-MAGEA3	Adenovirus vector Maraba virus	Melanoma associated antigen 3	Pembrolizumab	NSCLC	Phase I / II	2017–2020	Turnstone Biologics, Corp
NCT 02,043,665	CVA21	Coxsackie virus	None (CAVATAK)	Pembrolizumab	NSCLC	Phase I	2013–2020	Viralytics
NCT 03,647,163	VSV-IFNβ-NIS	Vesicular Stomatitis virus (VSV)	Interferon-beta (IFNβ) and the sodium iodide symporter (NIS)	Pembrolizumab	NSCLC	Phase I / II	2019–2021	Vyriad, Inc
NCT 00,861,627	REOLYSIN	Reovirus Serotype 3—Dearing Strain	KRAS/EGFR	Carboplatin Paclitaxel	NSCLC	Phase II	2009–2015	Oncolytics Biotech
NCT 01,708,993	REOLYSIN	Reovirus Serotype 3—Dearing Strain	KRAS/EGFR	Pemetrexed Docetaxel	NSCLC	Phase II	2012–2016	Canadian Cancer Trials Group
NCT 01,017,601	(NTX-010) Seneca Valley virus-001	Seneca virus	NA	NA	SCLC	Phase II	2010–2013	Alliance for Clinical Trials in Oncology
NCT 01,574,729	rAd-p53	Adenovirus	p53	surgery	NSCLC	Phase II	2012–2015	Shenzhen SiBiono GeneTech Co.,Ltd
NCT 03,004,183	ADV/HSV-tk	Herpes simplex virus	thymidine kinase	SBRT Pembrolizumab	NSCLC	Phase II	2017–2022	The Methodist Hospital Research Institute
NCT 02,831,933	ADV/HSV-tk	Herpes simplex virus	thymidine kinase	SBRT Nivolumab	NSCLC	Phase II	2017–2020	Eric Bernicker, MD

### Targeted antibodies for lung cancer

Scientists have well exploited the capability of the antibodies to specifically target tumor antigens on cancer cells and created a plethora of targeted antibodies to impair tumor cell activities. They can be broadly classified into three categories: (i) mAbs, (ii) antibody–drug conjugates (ADCs) (iii) bispecific antibodies (Fig. 3D). A few mAbs that received FDA approval for the treatment of NSCLC in the preceding two decades are—Cetuximab, Bevacizumab, Nivolumab, and Pembrolizumab (Table 2). Cetuximab is an anti-EGFR mAb that shows specific binding to the extracellular domain of EGFR and disrupts its receptor tyrosine kinase (RTK)-associated downstream proliferative activity. It has furnished positive responses in various combination therapy. Other efficacious anti-EGFR mAbs under evaluation in NSCLC are Necitumumab, Nimotuzumab, and Ficlatuzumab [141–144]. An anti-VEGF mAb Bevacizumab that showed anti-angiogenic potential to inhibit tumor growth was the first to receive FDA approval and is discussed in other sections. Another approved anti-VEGF mAb is ramucirumab which showed significant promise in combination therapy for NSCLC [145].

**Table 2:** Targeted antibodies for lung cancer therapy

Targeted Antibodies	Lung cancer type	Related Molecule	Target / Bioactivity
**Monoclonal Antibodies (MABs)**	**NSCLC**	Cetuximab	Anti-EGFR
		Necitumumab	Anti-EGFR
		Nimotuzumab	Anti-EGFR
		Ficlatuzumab	Anti-EGFR
		Bevacizumab	Anti-VEGF
		Ramucirumab	Anti-VEGF/VEGFR2
		Nivolumab	Anti-PD-1
		Pembrolizumab	Anti-PD-1
		Ipilimumab	Anti-CTLA-4
		Tremelimumab	Anti-CTLA-4
		Denosumab	Anti-RANKL
		Figitumumab	Anti-IGF-1R
	**SCLC**	Tarextumab	Anti-Notch2 / Notch3
		Tucotuzumab	Anti-EpCAM
		Bec2	Anti-GD3
**Antibody–Drug Conjugate (ADC)**	**SCLC**	Rovalpituzumab tesirine	Anti-DLL3
		Sacituzumab govitecan	Anti-Trop-2
		Lorvotuzumab mertansine	Anti-CD56
	**NSCLC**	Ado-Trastuzumab emtansine	Anti-HER2
		Telisotuzumab vedotin	Anti-cMET
		Enapotamab vedotin	Anti-AXL
**Bispecific antibodies**	**NSCLC**	Amivantamab	Anti-EGFR, Anti-MET

Immune checkpoint inhibitor (ICI)-based targeting antibodies prevent tumor cells from being attacked by immune system components ready to combat them. Among them, Nivolumab and Pembrolizumab are anti-PD-1 mAbs currently used in clinics. Also, two other anti-CTLA-4 mAbs undergoing rigorous evaluation are Ipilimumab and Tremelimumab. Their anticancer activities are elaborately discussed in other sections. Denosumab, an anti-RANKL (receptor activator of nuclear factor-kappa B ligand) mAb, showed efficacy in metastatic lung cancer inhibition in a phase III study [146]. Phase I and II study with Figitumumab, a fully humanized anti-IGF-1R MAB, as first-line therapy combined with chemotherapy showed considerable promise, but a phase III trial was discontinued [147]. For SCLC, efforts have also been directed to develop mAbs such as Tarextumab (anti-Notch 2 / Notch 3), Tucotuzumab (anti-EpCAM), and Bec2 (anti-GD3), which furnished positive outcomes in various clinical trials [148–150]. Nonetheless, to further enhance the efficacies of the mAbs, tripartite ADC has been synthesized where a potent cytotoxin is conjugated to mAbs via a covalent linker. Few of them are already commercialized, and many are undergoing different phases of clinical trials [151]. For refractory and metastatic SCLC, ADCs such as Rovalpituzumab tesirine (anti-DLL3), Sacituzumab govitecan (anti-Trop-2), lorvotuzumab mertansine (anti-CD56) is undergoing phase I/II clinical trials and are showing encouraging results [152–154]. Recent studies did not find an apparent efficacy with Rovalpituzumab tesirine in SCLC patient trials [155]. For NSCLC, various ADCs such as Ado-trastuzumab emtansine (anti-HER2), Telisotuzumab vedotin (anti-cMET), Enapotamab vedotin (anti-AXL) are under development and showing promising outcomes [156–158]. The third category of antibody-based targeted cancer therapy, referred to as bispecific T cell engagers or bispecific antibodies (BiTEs), are developed by fusing two front-end regions of two antibodies. One of its categories, Amivantamab (anti-EGFR, anti-MET), is now approved for lung cancer treatment [159]. Considering the clinical evidence accumulated in recent times, we envisage that many of the targeted antibodies will be approved in the near future.

### Immune checkpoint inhibitors and NSCLC

Inhibition of immune-checkpoint proteins by blocking the CTLA-4, PD-1, and PD-L1 has been the most successful immunotherapeutic strategy in NSCLC. Ipilimumab is a fully-humanized mAb capable of neutralizing the CTLA-4, thus enabling CTL activity and sustaining immune responses mostly by depletion of regulatory T cells (Tregs) that demonstrate high levels of CTLA-4 expression [160]. Lynch et al. demonstrated in a preliminary phase II study (CA184-041) that Ipilimumab, in combination with chemotherapy in the first-line treatment for metastatic stage IIIB/IV NSCLC showed an enhancement in immune-related progression-free survival (irPFS) compared to only chemotherapy, without significant added toxicities [161, 162]. Its combinatorial application with erlotinib, crizotinib, and nivolumab has also been studied in a phase Ib non-randomized clinical trial (NCT01998126) for EGFR and ALK translocation-positive stage IV NSCLC that was completed in 2018 [163]. MS was not reached, and an excessive toxicity profile led to the termination of the study. Few more studies that combine Ipilimumab with radiation (NCT02239900, phase I/II, randomized, 143 participants and NCT02221739, phase I/II, 39 participants) and PD-1 antibody (discussed underneath) are currently ongoing. Tremelimumab is a fully human mAb that explicitly targets human anti-CTLA4. After it recorded an initial failure in a phase II randomized trial (*n* = 87) when administered in patients with pre-treated advanced-stage NSCLC, recently it came forth from the phase III NEPTUNE trial (NCT02542293, phase III, randomized, 953 participants) that a combination of Tremelimumab plus Imfinzi (Durvalumab, anti-PD-L1 antibody) performed no better than standard chemotherapy at extending the survival of people with metastatic NSCLC [164].

On the contrary, antibodies targeting the PD-1 protein have shown greater therapeutic promise in NSCLC. While CTLA-4 pathway inhibitors increase the infiltration and repertoire of tumor-specific T cells, PD-L1/PD-1 inhibitors work by preventing the inhibition of T-cell functions. Nivolumab (brand name Opdivo) is a fully human anti-PD-1 IgG4 mAb that specifically targets the human PD-1 protein. Preliminary data obtained from a phase I clinical trial (NCT00730639, non-randomized, 395 participants) of Nivolumab was used in advanced or recurrent malignant patients, including NSCLC, spearheaded three key trials that presented their results in 2015 [165–167]. The phase II, single-arm CheckMate 063 trial (NCT01721759, 117 participants with advanced, refractory squamous NSCLC) demonstrated that intravenous administration of Nivolumab (3 mg/kg) every two weeks resulted in 14.5% (17 of 117) patients achieving an objective response (OR), the primary endpoint for the investigation while 26% of (30 of 117) patients showed stable disease [165]. 17% of patients were reported to have developed grade 3/4 adverse events (AE), the most frequent of which were: pneumonitis (3%), diarrhea (3%), and fatigue (4%). CheckMate 057 (NCT01673867, randomized, phase III study, 272 participants) evaluated Nivolumab's effectiveness and safety in patients with disease progression during or after first-line chemotherapy for patients with advanced squamous NSCLC and was compared to docetaxel [167]. Although the MS for Nivolumab was higher than with docetaxel (9.2 vs. 6.0 months), but PD-L1 expression was of neither predictive nor prognostic benefit. The phase III CheckMate 017 trial (NCT01642004, open-label, 352 participants) investigated the effect of Nivolumab (at 3 mg/kg every two weeks) as compared to docetaxel (at 75 mg/m^2^ every three weeks) in patients with IIIB/IV non-squamous NSCLC that advanced during or after first-line chemotherapy [166]. The MS in the Nivolumab group was 12.2 months, compared to 9.4 months in the docetaxel group. Nivolumab did not have the edge over docetaxel in terms of PFS; the study is ongoing. However, Nivolumab revealed a better efficacy than docetaxel across all categories determined by the degree of PD-L1 expression on the tumor cell membrane. Also, treatment-related severe AEs were observed in 10% of the patients treated with Nivolumab, against 54% with docetaxel. The FDA approved Nivolumab as the first anti-PD-1 drug to treat pre-treated advanced or metastatic NSCLC. The scheme of immunotherapy treatment in NSCLC patients is shown in Fig. 3.

Moreover, additional clinical trials like CheckMate 012 are currently underway to assess the efficacy of Nivolumab with or without Ipilimumab in first-line settings for advanced NSCLC. In the CheckMate 012 trial (NCT01454102, phase I, open-label, 472 participants to date), Nivolumab was initially tested as a monotherapy in first-line advanced stage IIIB/IV NSCLC, which resulted in a 23% (12 out of 52) ORR in newly diagnosed advanced NSCLC patients and the investigators found four patients with continuing complete responses [168]. The ORR was 28% (9 out of 32) in subjects with tumors expressing PD-L1 and 14% (2 of 14) in subjects with no detectable PD-L1 expression. Later, when tested in combination with Ipilimumab or another platinum-based chemotherapy cohort (*n* = 56), the trial showed a significant rate (45%) of AEs for which treatment discontinuation occurred in significant numbers [165]. ORR was achieved regardless of tumor PD-L1 expression and the respective ORRs were 33%, 47%, 47%, and 43% for Nivolumab (10 mg/kg) with gemcitabine/cisplatin, Nivolumab (10 mg/kg) along with pemetrexed/cisplatin, Nivolumab (10 mg/kg) in combination with paclitaxel/carboplatin, and Nivolumab (5 mg/kg) plus paclitaxel/carboplatin in this study. CheckMate 277 trial (NCT02477826, randomized, open-label, phase III, 2748 participants) evaluated Nivolumab or Nivolumab plus Ipilimumab, or Nivolumab in combination with platinum-doublet chemotherapy to platinum doublet chemotherapy in PD-L1-defined previously untreated NSCLC [169]. The results exhibited positive outcomes regarding OS with nivolumab plus ipilimumab compared to chemotherapy in patients irrespective of the expression of PD-L1 [170]. CheckMate 9LA presented an interesting improvement in the OS for advanced NSCLC patients with two cycles of chemotherapy in combination with Nivolumab and Ipilimumab [170, 171]. CheckMate 227 and CheckMate 9LA prompted a chemo-free doublet immunotherapy approach and improved the overall OS regardless of the patient’s PD-L1 profile.

Pembrolizumab (MK-3475) is another high-affinity humanized IgG4 mAb that targets the PD-1 protein. The drug's safety profile and therapeutic efficacy in NSCLC were initially assessed in the phase I clinical KEYNOTE-001 study (NCT01295827, phase I, randomized, open-label, 1260 participants), which demonstrated durable antitumor activity in advanced-stage NSCLC patients [172, 173]. Patients received Pembrolizumab at either 2 mg/kg (*n* = 55) or 10 mg/kg (*n* = 238) every 3 weeks or 10 mg/kg (*n* = 156) every 2 weeks and response was evaluated every 9 weeks. ORRs for the doses were 15% [95% CI, 7%-28%] at 2 mg/kg every three weeks, 25% (95% CI,18%-33%) at 10 mg/kg every three weeks, and 21% (95% CI,14%-30%) at 10 mg/kg every two weeks respectively, which suggest the use of a 2 mg/kg Pembrolizumab every three weeks as the optimum dosage in patients with previously treated, advanced NSCLC. Subsequently, KEYNOTE-010 (NCT01905657, randomized, phase II/III study, 1034 participants) compared the dosage gradient of Pembrolizumab with a fixed dose of docetaxel in patients who were pre-treated with advanced NSCLC (expressing PD-L1 ≥ 1%), keeping OS and PFS as the primary endpoints. Although the MS was considerably extended for Pembrolizumab 2 mg/kg as compared to docetaxel [HR 0.71, 95% confidence interval (CI) 0.58–0.88] and for Pembrolizumab 10 mg/kg when compared to docetaxel (HR 0.61, 95% CI 0.49–0.75), no statistically significant variance in the overall median PFS was observed. Notably, PFS was significantly longer with Pembrolizumab in patients whose tumor cells express at least 50% PD-L1. Following the results, the FDA approved Pembrolizumab to treat patients with advanced PD-L1 expressing NSCLC whose disease had worsened following chemotherapy in October 2015. Again, in the phase III KEYNOTE-024 trial (NCT02142738) that assessed the effectiveness of Pembrolizumab as first-line therapy compared to different chemotherapy regimens for metastatic treatment-naive NSCLC, the drug once again proved its advantage over only chemotherapy with significantly longer PFS (10.3 vs. 6.0 months; HR 0.50; *P* < 0.001) and OS (HR 0.60; 95% CI, 0.41 to 0.89; *P* = 0.005) in patients receiving the drug. Moreover, the Pembrolizumab group showed a response rate of 44.8% versus 27.8% in the chemotherapy-treated group, and severe AEs were reported to occur in ~ 26.6% of the Pembrolizumab group patients versus 56.6% of the patients in the chemotherapy-treated group. This led to the FDA approval of the drug for the first-line treatment of advanced metastatic NSCLC patients with high tumor PD-L1 expression (at least 50% tumor cells) [174].

The third group of check-point inhibitors target PD-L1 to inhibit the molecular interaction between PD-L1 and PD-1 or the molecular contact between PD-L1 and B7.1 (a T cell-specific inhibitory receptor). Durvalumab, Atezolizumab, and Avelumab are three fully-humanized anti-IgG1 mAbs that comprise this class of drugs. Durvalumab (MEDI-4736) was tested by a phase I/II trial in stage IIIB/IV NSCLC and other solid tumors. Durvalumab (10 mg/kg) was administered every two weeks for up to one year to treatment-naïve advanced NSCLC patients. The ORR was 25%, and the disease control rate was 56% with ≥ 12 weeks of follow-up, and grade ≥ 3 drug-related AEs (most frequent being diarrhea) were reported in 9% of patients [175]. Taking forward these encouraging outcomes, the efficacy of Durvalumab is being evaluated in trials for various aspects such as monotherapy (NCT02087423) after concurrent chemo-radiotherapy in stage III NSCLC (NCT02125461) also adjuvant therapy in patients with stage IB to IIIA NSCLC (BR31 trial; NCT02273375).

Again, a combinatorial study with Durvalumab and Tremelimumab was initiated to evaluate the postulate stating that co-inhibition of PD-1/PD-L1 and CTLA-4 may evoke synergy in immunotherapy in patients with advanced NSCLC [176]. The outcome demonstrated that 36% of patients developed AEs, and 23% of patients achieved ORR in a combined Tremelimumab 1 mg/kg cohort. Many phase II/III trials, including third-line ARCTIC (NCT02352948), the first-line MYSTIC (NCT02453282), and NEPTUNE (NCT02542293), have been commenced using a combination strategy of immuno-therapeutics. In the case of Atezolizumab (MPDL3280A), after a phase I study confirmed its efficacy for treatment in NSCLC (ORR of 23%, *n* = 53), especially in patients with tumor cell PD-L1 expression, a single-arm phase II study (BIRCH, NCT02031458, open-label, 667 participants) in PD-L1 selected (tumors or immune cells in TME) advanced NSCLC was initiated with ORR being the primary endpoint [177, 178]. The patients received Atezolizumab (1,200 mg) intravenously every three weeks and were distributed into three cohorts: first-line (cohort 1, with no prior chemotherapy; *n* = 139); second line (cohort 2, with one prior platinum chemotherapy; *n* = 268); and third-line or higher (cohort 3, with at least two prior chemotherapies of which one is platinum-based; *n* = 252). It was observed that BIRCH achieved its primary goal by exhibiting a significant increase in ORR (18% to 22% for the three cohorts) in Atezolizumab treated patients compared to historical controls, and most of the responses are ongoing. Also, the MS (minimum of 20 months follow-up) for cohort 1 was 23.5 months, cohort two was 15.5, and cohort 3 was 13.2 months. Thus, the trial showcased responses with good tolerability for Atezolizumab monotherapy in advanced-stage NSCLC patients with PD-L1 selected tumors.

**Fig. 6:**
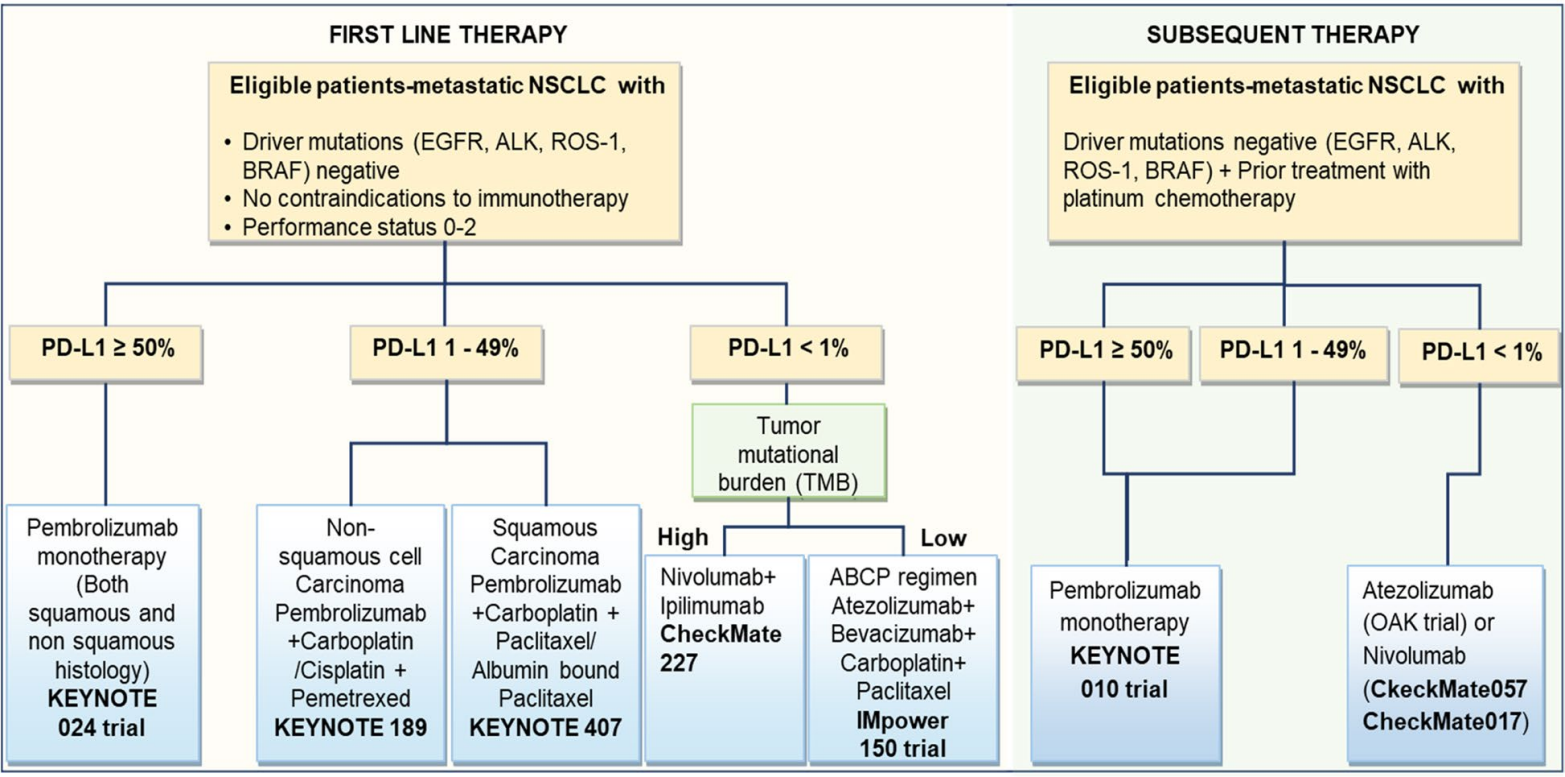
Scheme of treatment with immunotherapy in NSCLC. The algorithm helps the physician decide on the available treatments for different types of lung cancers. Their consensus sequencing techniques. PD-L1 testing and histological subtype determination should be done by a multidisciplinary team for all patients. The role of driver mutation, as shown, is important in determining treatment modalities

Another phase II trial (POPLAR, NCT01903993, randomized, open-label, 287 participants) assessed the safety and efficacy of Atezolizumab-based immunotherapy compared to docetaxel therapy in NSCLC patients pre-treated with platinum-based chemotherapy [179]. Volunteers were randomly distributed (1:1) into two groups where one group received intravenous Atezolizumab (1,200 mg) and the other received docetaxel (75 mg/m^2^), both for three weeks intervals. As observed at the primary endpoint, the OS was improved for Atezolizumab (12.6 months) compared to Docetaxel (9.7 months, HR 0.73, *P* = 0.04). This can be attributed to the alteration in PD-L1 expression. Also, a diminution of treatment-induced grade 3/4 AEs in patients treated with Atezolizumab (11%) versus the docetaxel treated group (39%) strongly advocated for the possible benefits of Atezolizumab in NSCLC patients who received previous treatments [179]. Finally, the propitious outcomes obtained in the BIRCH and POPLAR trials led to the FDA approval of Atezolizumab for patients with advanced NSCLC. The NCT01846416 trial tested the drug as monotherapy for PD-L1 positive patients with advanced non-metastatic NSCLC. The third one, Avelumab, has demonstrated reasonable clinical efficacy and safety profile in NSCLC patients untreated previously and unselected for PD-L1 expression through NCT01772004 and NCT02395172 [180]. However, further investigations are required to prescribe the drug for practical applicability. NSCLC tumor PD-L1 expression has become an essential determinant of clinical pathology and frontline treatment. A summary of the treatment strategy in different crucial clinical trials is shown in Fig. 6. Though the studies help draw certain conclusions, more studies are needed to draw meaningful comparisons to improve NSCLC immunotherapy for both PD-L1 high and low populations.

Anti-T-cell immunoreceptor with Ig and ITIM domain (TIGIT) checkpoint inhibitor Tiragolumab suppresses innate and adaptive immunity in PD-L1-positive metastatic NSCLC and has received the Breakthrough Therapy Designation (BTD) by FDA [181]. LAG3 and FGL1 expression promote tumor development by suppressing the immune system and are amongst the most promising immune checkpoints. LAG3 accumulates on CD4 + and CD25-T cell surfaces in TILs and is also identified in the cytoplasm of NSCLC cells, while FGL1 is identified in NSCLC cell's cytoplasm [182]. LAG-3 antibody (liratrimab) may have therapeutic utility as a third ICI route after PD-1 and CTLA-4. A soluble LAG-3 protein eftilagimod alpha (Efti; IMP321) is also undergoing a phase II study (TACTI-002) (NCT03625323) in combination with pembrolizumab on metastatic NSCLC patients [183]. Nobel ICPs are emerging and need to be investigated as single agents and combinations. The current immunotherapy pipelines have concentrated on functional, cellular, and molecular readouts but lack mechanistic knowledge of immunotherapy targets.

Classification of lung cancer progression patterns and grading and updating the Common Terminology Criteria for Adverse Events (CTCAE) in relation to ICIs and associated diagnostic markers can help better design the clinical trial endpoints. Understanding the immune-related adverse events (irAEs) concerning different ICIs and therapeutic combinations can help plan better neutralizing mechanisms. Using appropriate pre-clinical models, including human-specific organoid models, humanized mice, and rigorous pharmacokinetic/ pharmacodynamic analysis, can help reduce clinical trial failures. Further investigation regarding the ICI treatment duration, rechallenge, and dose-ranging need attention. Advances in single-cell genomics, multiplex immunofluorescence, and multi-omic platforms encourage therapeutic discovery by finding and evaluating new immunotherapy targets. AI-based algorithms are currently being considered for radiological, pathological, and diagnostic data analysis and can help build successful immunotherapies and data-driven medication schedules and combinations. Recently Sun et al. developed a radiomics-based biomarker of tumor-infiltrating CD8 cells on patients in a phase I study with anti-PD-1 or PD-L1 monotherapy. They proposed imaging biomarkers may assess CD8 cell count, correlate with tumor immune profile, and predict immunotherapy patient outcomes in many solid tumors, including lung cancer [184]. The deployment of artificial intelligence in prospective clinical trials is constrained by various obstacles, such as the lack of a high-quality training data set, validation data set, code sharing, and transparency, despite the technology's innovation and significant promise [185].

### Immunotherapy in SCLC

Lung SCLC, mostly begotten by cigarette smoking, is an abjectly differentiated, fast-growing, high-grade, malignant epithelial cell carcinoma originating from neuroendocrine cells within the bronchial airways. SCLC cells are morphologically diverse, with poorly defined borders, tiny cytosol, granulated nuclear chromatin, and absence or unobtrusive nucleoli with a high mitotic count [186, 187]. 5% of SCLCs can be originated from extrapulmonary regions, including the nasopharynx, GI tract, and genitourinary tract [188–190]. However, SCLCs of pulmonary and extra-pulmonary origins have similar clinical and biological features characterized by rapid growth and early widespread metastasis [191–194]. About 70% of patients with ED-SCLC have apparent metastases at the time of diagnosis. The remaining 30% have LD-SCLC, defined by tumors confined to the hemithorax [195, 196]. This inevitably results in a poor prognosis for the patients, with MS being 15–20 months and 8–13 months for LD and ED-SCLC, respectively [195]. Similarly, SR on average over 5 years interval is 10%–13% and 1%–2% for LD and ED-SCLC patients, respectively [195, 197].

The aggressive nature of SCLC can be ascribed to its high TMB, which includes the bi-allelic deactivation of tumor suppressor genes, such as p53 and Rb1, in nearly all tumors incidences [198]. High TMB-associated generation of a higher number of neoantigens allows an enhanced presentation to the T cells leading to a heightened immunological response and may be exploited to create efficient immunotherapeutics. [199, 200]. SCLC has long been considered immunogenic due to its association with paraneoplastic disorders, such as Lambert-Eaton myasthenic syndrome (LEMS) in patients. This results from immune responses directed against specific antigenic targets (HuD, HuC, and Hel-N1) expressed on both SCLC and normal nerve cells [201–205]. Intriguingly, SCLC patients having LEMS are likely to obtain a better prognosis, which can be attributed to the phenomenon that the immune response directed against the nervous system can also target the tumor cells [203]. Also, better OS was observed in patients whose tumors were infiltrated with more CD45^+^ T cells, independent of stage and performance status. A correlation between higher effector-to-regulatory T-cell ratios and prolonged OS was established [202, 204, 205]. Recent developments have been remarkable in immunotherapy-based approaches for SCLC, such as ICIs, antigen-specific vaccines, and tumor vaccines, fostering hope for a general increase in the SR, OS, and patient quality of life.

### Antigen-specific vaccines and SCLC

A vaccine-based approach has often been considered ideal for SCLC patients, especially those who have recently completed chemotherapy cycles, due to its minimal toxicity potential. Almost four decades ago, gangliosides and glycolipids were identified as therapeutic targets for the treatment of melanoma [206, 207]. Soon it was noted that most SCLC cell lines express GD3, a glycosphingolipid (ceramide and oligosaccharide) or oligoglycosylceramide containing one or multiple sialic acids (i.e., n-acetylneuraminic acid), which prompted the evaluation of BEC2, the anti-idiotypic mAbs mimicking GD3 ganglioside in SCLC patients [208]. In a pilot trial conducted on 15 patients who were administered BEC-2 adjuvanted with BCG after completion of initial chemotherapy, the OS was enhanced by 21 months [209]. Further large-scale trials, sponsored by the European Organization for the Research and Treatment of Cancer, detected a 40% increase in survival. The combination has currently been licensed by ImClone Systems, USA, and Merck Oncology in Europe, Australia, and New Zealand.

Upon further investigations and trials, four other antigens—GM2, Globo H, fucosyl GM1, and polysialic acid emerged as antigenic targets for immunotherapy in SCLC based on immune-histochemical analyses of tumor samples [210]. Although the immunogenicity for GM2 and Globo H was well-understood in other forms of malignant cancers, fucosyl GM1 and polysialic acid were selectively expressed in SCLC alone [211–215]. All the antigens were tested as conjugates with KLH and QS-21 as the adjuvant for administration in patients who completed initial chemotherapy or radiation therapy. Additionally, It was ensured that the patients were not taking systemic corticosteroids and were free from any underlying immune deficiencies or peripheral sensory neuropathy greater than Grade 1 [206]. Although some immune-specific responses had been noted in patients administered with the antigens, trials with a bigger cohort are still needed to validate their efficacy in the prognosis for SCLC patients.

Another target antigen recently surfaced in the treatment of SCLC is p53. An initial trial with 29 ED-SCLC patients given the p53 vaccine plus chemotherapy showed a high overall response rate (ORR). Similarly, induction of immune response (40–50%) and tolerability of a dendritic cell-based vaccine with modified p53 (INGN-225) was evaluated in a phase I/II study [216]. However, the optimal treatment in all of the above cases may not suffice with vaccination alone and ought to be coupled with chemotherapy scrupulously [217]. Thus, further trials are required to verify the role and extent of antigen-specific vaccines as a potential therapeutic approach in patients with SCLC. In another innovative approach by Sakamoto et al., the efficacy of a personalized peptide vaccination (PPV) was evaluated in a phase II trial where a maximum of 4 HLA-matched peptide sequences were selected from a previously established IgG response-specific peptide library [218]. The PPV was subcutaneously administered, in which 46 patients were enrolled. Each of the four groups of patients had a MS of 466 (0; *n* = 5), 397 (1; *n* = 15), 401 (2; *n* = 12), and 107 days (3; *n* = 14), respectively, in terms of prior treatment regimens. After one and two vaccination rounds, peptide-specific IgG responses increased in 70% and 95% of patients. OS was considerably longer in individuals with increased IgG responses after the second immunization cycle (1237 vs. 382 days; *P* = 0.010) than in patients with enhanced IgG responses. Despite some positive outcomes in OS prolongation and immune rejuvenation, further evaluation of its efficacy in eventual randomized trials is necessary.

### Immune-checkpoint inhibitors for SCLC

Blocking the immune checkpoints with mAbs has gained significant attention as a promising therapeutic tool in oncology, including SCLC [5, 219]. While eliciting an antigen-specific T-cell response, costimulatory and co-inhibitory factors play a key role in immune regulation post-stimulation of the TCR [220]. After the TCR recognizes the antigenic peptides displayed by both the classes of MHC I & II molecules on the surface of antigen-presenting cells (APC), the entire T-cell activation process requires a second costimulatory signal generated by the costimulatory T-cell surface receptor CD28 that binds to B7 ligand subtypes CD80 and CD86 present on APCs surface [5, 221]. T-cell activation and subsequent immunological response are aided by the co-stimulation of CD28 with other related molecules, such as CD134 and CD137. Another fraction of molecules, viz., CTLA-4, PD-1, B7-H3, and B7x abate antigen-specific immune responses by restricting their magnitude and duration. These co-inhibition molecules are called “immune checkpoint proteins,” and inhibition of these protein pathways (immune-checkpoint inhibition) by blocking CTLA-4 and PD-L1 with mAbs, etc., have shown potential advances in cancer immunotherapy [219].

It has been postulated that PD-1 and its ligand on the SCLC cells may be involved in tumor cell growth inhibition. [222]. Pembrolizumab is an anti–PD-1 mAb designed to block the PD-1/PD-L1 pathway. To test the efficacy of this antibody, in the KEYNOTE-028 phase Ib trial, 24 patients with PD-L1^+^ ED-SCLC who had completed initial chemotherapy received Pembrolizumab. Although the study produced an ORR of 35%, with lasting responses over more than 16 weeks, the related AE rate was 53% [223]. In another study, using immunohistochemistry, Ishii et al. examined the expression of PD-L1 in 102 SCLC patients where 71.6% of volunteers expressed PD-L1, and its correlation with LD-SCLC was established. The results revealed that MS was 16.3 months in the PD-L1( +) subset and 7.3 months in the patients not expressing PD-L1 (*P* < 0.001) [224]. Few more prospective trials (NCT02359019 and NCT02403920) investigating Pembrolizumab with chemotherapy or Pembrolizumab with chemotherapy and radiotherapy for use in SCLC are ongoing. These clinical studies are trying to address the issue of limited treatment options for patients with metastatic SCLC who are on platinum-based chemotherapy, which may help SCLC patients.

Similarly, as previously mentioned, CTLA-4 is an immune checkpoint protein that is expressed on activated T cells, which is widely studied for its capacity to down-regulate T-cell activities [225]. With the development of fully human mAbs such as Ipilimumab, CTLA-4 has become an attractive therapeutic target for cancer. Of many recent clinical trials, a randomized phase II study has explored the combinatorial application of Ipilimumab with paclitaxel and carboplatin as the first-line treatment in ED-SCLC [226]. The outcome revealed that phased Ipilimumab improved the PFS compared to concurrent Ipilimumab. The OS of the two groups were 12.5 and 9.1 months respectively, with no significant difference (*P* = 0.13) [226]. Prompted by the outcomes, a phase III clinical trial (NCT01450761) of Ipilimumab along with chemotherapy compared to chemotherapy alone has commenced in ED-SCLC patients.

In addition, another phase I/II clinical trial (Checkmate 032, NCT01928394) evaluated the effectiveness of Nivolumab (an anti-PD-1 mAb) coupled with or without Ipilimumab in patients with limited-stage or extensive-stage SCLC relapse after at least one platinum-containing regimen [227]. The results presented that Nivolumab monotherapy and Nivolumab in combination with Ipilimumab exhibited anticancer activity with protracted responses and the adverse events were tolerable. More such trials are in-line. Thus, the constant efforts to improvise these strategies give us the hope of better future outcomes for SCLC prognosis and treatment.

## Combination immunotherapy approaches

Because the immune response is dynamic, evidence suggests that combination therapy may improve cancer patient survival compared to monotherapy. Anti-angiogenic agents, chemotherapy, radiation therapy, and T-cell modulation are investigated in combination with immunotherapy. The arsenal of immunotherapeutics is rapidly gaining ground while unveiling new challenges. Finding the optimal therapy combinations, doses, and order of administration in specific cancer genotypes needs to be investigated. Other challenges are irAEs, finding suitable biomarkers for immunotherapy response, resistance to immunotherapy, and making the non-responders benefit from combination therapy. Understanding the tumor type, TME-mediated immunosuppression, immune profile of the tumor, and tumor genotypic profiling may help address some of the challenges. AI can analyze and categorize input data and produce models to anticipate molecular interactions, the efficiency of combination therapies, and predict poor prognosis associated genotypes and needs further attention. The forthcoming section summarizes the present state of combination therapy and clinical application.

### Immunotherapy with Anti-angiogenic agents for targeting NSCLC

There is no dearth of preclinical evidence for the general validity of the proposition that angiogenesis is crucial for tumor growth, and it possesses a convoluted relation with tumor immunity, i.e., antiangiogenic compounds can invigorate the immune system while cancer immunotherapy turns to be antiangiogenic [228–230]. More importantly, a combination of these two can have a synergistic impact on inhibiting tumor growth. The immunosuppressive TME, comprising VEGF as a major modulator of immune response, endorses tumor cells towards evasion of immune surveillance [231]. VEGF (i) blocks lymphocyte trafficking across activated tumor endothelium by inducing clustering defects at the endothelial cell surface, (ii) inhibits tumor infiltration of the T cells via upregulation of the Fas ligand, (iii) induces proliferation in immune suppressors, viz., Tregs and myeloid-derived suppressor cells (MDSCs), etc. [232–234]. Antiangiogenic therapy normalizes the tumor blood vessels decreasing interstitial fluid pressure, thereby enhancing drug penetration within the tumor and synergizing with chemotherapy and immunotherapy [235, 236]. A study comprising 125 advanced NSCLC patients showed that anti-VEGF therapy, bevacizumab, mediated metabolic changes of the tumor through the LKB1/AMPK pathway, which correlates with increased survival [237]. Many preclinical and clinical data also hint at the possibility of synergy between immunotherapy and anti-angiogenic therapy in NSCLC, enhancing the potential of both [238]. For instance, using an in vivo NSCLC model, Tao et al. showed that immunotherapy in combination with bevacizumab inhibits tumor growth synergistically, and the approach holds promise for clinical translation [239]. Multiple clinical trials are ongoing with this combinatorial approach and, hopefully, will move from bench side to bedside shortly [240, 241].

### Chemo-immunotherapy in lung cancer

As discussed in the earlier sections, inhibition of the PD-1 receptor or PD-L1 is the mainstay of action of current immunotherapy agents in NSCLC and SCLC. Nonetheless, the combinatorial applications of immunotherapeutic agents with Chemotherapy drugs have emerged as a much-practiced method for managing progressive disease. Below we discuss the same elaborately. Few more trials like the NCT02486718 (IMpower010), NCT02657434 (IMpower132), NCT02409342 (IMpower110), NCT02367781 (IMpower130), and NCT02366143 (IMpower150) are ongoing to test the efficacy of Atezolizumab with chemotherapy. Furthermore, several combination treatments of the drug with other intervention approaches such as the MEK inhibitor cobimetinib (NCT01988896), the tyrosine kinase inhibitor drug cabozantinib (NCT03170960), the anti-VEGF-A humanized monoclonal IgG Bevacizumab (NCT03836066, NCT03616691), etc. are also being evaluated.

### Chemo-immunotherapy in NSCLC

The primary immunotherapy agents employed are Pembrolizumab, Atezolizumab, Durvalumab, Nivolumab, and Ipilimumab. Figure 6 briefly depicts the strategy taken for the treatment with immunotherapy in NSCLC.

#### Pembrolizumab

It is an immune checkpoint inhibitor for the PD-1 receptor, which improves anti-tumor immunity. As per treatment guidelines, every patient with metastatic NSCLC should undergo IHC testing for PD-L1 expression and other driver mutations like EGFR, ALK, ROS-1, and BRAF before starting treatment [242].

##### As first-line therapy

In eligible patients (Performance status 0–2 and no contraindication for immunotherapy) with metastatic NSCLC irrespective of histology with PD-L1 level ≥ 50% and with none of the driver mutations (EGFR, ALK, ROS-1, BRAF), Pembrolizumab is recommended as first-line monotherapy. Patients with PD-L1 expression 1–49% and all driver mutations remaining negative are recommended Pembrolizumab plus chemotherapy as the first-line therapy. For Non-Squamous/Adenocarcinoma, combination chemotherapy of Carboplatin or Cisplatin with Pemetrexed is preferred with Pembrolizumab. In patients with squamous cell histology, combination chemotherapy of Carboplatin and Paclitaxel/Albumin bound paclitaxel is preferred with Pembrolizumab [243]. KEYNOTE-024 trial aimed at comparing Pembrolizumab monotherapy vs. platinum-based chemotherapy as first-line therapy for NSCLC patients with PD-L1 expression level ≥ 50% and driver mutations negative. It showed an improved response rate and MS with Pembrolizumab monotherapy (30.0 vs. 14.2 months) [174]. KEYNOTE-189, a randomized phase III trial with metastatic non-squamous NSCLC patients, compared Pembrolizumab and Carboplatin or Cisplatin and Pemetrexed vs. chemotherapy alone. It showed better OS at 1 year with Pembrolizumab plus chemotherapy (69.2% vs. 49.4%) [244]. KEYNOTE-407, a randomized phase III trial performed with metastatic squamous cell NSCLC patients, also evaluated the efficacy of Pembrolizumab together with Carboplatin and Paclitaxelor Albumin bound Paclitaxel. It resulted with improved MS in case of Pembrolizumab plus chemotherapy (15.9 vs. 11.3 months) [245].

##### As subsequent therapy

KEYNOTE 010, a phase III randomized trial carried out with advanced NSCLC patients who were previously treated and PD-L1 positive ≥ 1% and driver mutation-negative compared Pembrolizumab with chemotherapy (Docetaxel). It showed more prolonged overall survival for Pembrolizumab [246].

#### Atezolizumab

##### As first-line therapy

It is recommended as first-line therapy with ABCP regimen (Atezolizumab + Bevacizumab + Carboplatin + Paclitaxel) in patients with metastatic non-squamous NSCLC [247]. IMpower 150, a phase III randomized trial, evaluated the efficacy of first-line therapy with the ABCP regimen in patients with metastatic non-squamous NSCLC vs. Bevacizumab and chemotherapy, resulting in better MS (19.2 vs. 14.7 months) [248].

##### As subsequent therapy

Patients with metastatic NSCLC, during or after systemic therapy, are recommended with Atezolizumab as subsequent therapy. In a phase III randomized trial, OAK, Atezolizumab, and Docetaxel were compared as subsequent therapy in metastatic NSCLC patients, where Atezolizumab exhibited a better OS. [249, 250].

#### Durvalumab

PACIFIC, In a phase III randomized trial, PACIFIC, the efficacy of adjuvant treatment with Durvalumab was estimated as consolidation immunotherapy compared to placebo with unresectable stage III NSCLC after receiving treatment with concurrent treatment chemoradiation. The result showed increased OS and PFS (17.2 vs. 5.6 months) after Durvalumab consolidation therapy [251].

#### Nivolumab with or without ipilimumab

Nivolumab and Ipilimumab are immune checkpoint inhibitors with a complementary mechanism of action on T-cells where Nivolumab inhibits PD-1 receptors, and Ipilimumab is a human CTLA-4 blocking antibody.

##### As first-line therapy

In a phase III randomized trial, CheckMate 227, first-line Nivolumab/Ipilimumab was evaluated in comparison to Nivolumab monotherapy and chemotherapy in patients with metastatic NSCLC with high TMB (TMB ≥ 10 mutations per megabase) and negative for any driver mutations. It reports a better median PFS for Nivolumab/Ipilimumab (7.2 vs. 5.5 months) [169].

##### As subsequent therapy

In CheckMate 057, a phase III randomized trial, the efficacy of Nivolumab was compared with Docetaxel as subsequent therapy for metastatic non-squamous NSCLC patients during or after first-line chemotherapy. The trial reports better MS in the Nivolumab arm (12.2 vs. 9.4 months) [166]. In another phase III randomized trial, CheckMate 017, the efficacy of Nivolumab was also compared with Docetaxel as subsequent therapy for metastatic squamous cell NSCLC patients who had disease progression after chemotherapy. The report showed better MS with Nivolumab (9.2 vs. 6 months) [167].

### Chemo-immunotherapy in SCLC

#### Immunotherapy is only indicated in patients with extensive-stage SCLC

##### As first-line therapy

IMpower 133, a phase III randomized trial with ED-SCLC patients, adding Atezolizumab to Platinum and etoposide demonstrates improved MS (12.3 vs. 10.3 months) than platinum plus etoposide, which was standard of care for many years [252]. The observed survival advantage in IMpower 133 trial is independent of the PD-L1 expression and the TMB levels. Atezolizumab, in combination with the etoposide-platinum, is now recommended as first-line therapy by the NCCN panel for patients with ED-SCLC [243, 253]. CASPIAN trial is another randomized phase III trial that evaluated Durvalumab with etoposide-platinum in comparison to etoposide-platinum only as first-line therapy for ED-SCLC patients. Durvalumab, in combination with chemotherapy, significantly improved the OS [254].

##### As subsequent therapy

Pembrolizumab is recommended as subsequent therapy in patients with relapsed SCLC regardless of PD-L1 level following the recent analysis of two studies, KEYNOTE 028 and KEYNOTE 158 [255]. Nivolumab alone or with Ipilimumab is recommended as new subsequent therapy for patients who have relapsed within 6 months after first-line therapy. Checkmate 331, a randomized phase III trial, suggested using Nivolumab monotherapy. Also, CheckMate 032, a phase II trial, reported using Nivolumab in combination with Ipilimumab in relapsed patients [227]. Patients who progress after receiving first-line Atezolizumab should not be treated with any other immunotherapy.

### Radiation and immunotherapy

Though few trials are ongoing to evaluate the efficacy of any immune CKIs for use as concurrent therapy with radiation in cases of early or locally advanced lung cancer, there is no definite recommendation to date [256]. After the PACIFIC trial, Durvalumab has been recently recommended as consolidation immunotherapy in patients of unresectable stage III NSCLC post-treatment with concurrent chemoradiation [166].

### Immunomodulatory nanomedicine for use in lung cancer

Our prior discussion shows that immune stimulation is required for cancer treatment to detect and annihilate the non-self-antigens and create a memory effect as a future remedy. While a myriad of immunotherapeutics has obtained commendable results in the treatment of various cancers, they also faced some daunting challenges such as low water solubility, poor pharmacokinetic profiles viz., less absorption, less accumulation in the tumor region, thereby less bioactivity after prolonged circulation, and enhanced immune-mediated off-target toxicity [257, 258]. To our intrigue, nanotechnology, with all its propitious facets, is capable of addressing the existing and ensuing issues, thereby accomplishing the expected achievement level in terms of therapeutic benefits [259–262]. With a better understanding of the tumor microenvironment, smart stimuli-responsive nanocarriers are being developed to take advantage of acidic pH, hypoxia, increased ATP synthesis, changed redox state of cancer cells, and other factors [263]. It turns out that nanoparticles enhance the benefits of cancer immunotherapy via (i) providing protection to antigens and adjuvants, (ii) simultaneous delivery to the APC (iii) TME reprogramming to recommence immune surveillance.

As of now, a multitude of nanomaterials have demonstrated their immunomodulatory potential in pre-clinical, and few of them have undergone various phases of clinical trials [264, 265]. For instance, a liposomal cancer vaccine (L-BLP25) was developed by Oncothyreon Canada Inc., where the antigen tecemotide (carcinoma-associated human MUC-1) and an adjuvant 3-*O*-Deacyl-4’-monophosphoryl lipid A (MPL) were inserted into the lipid bilayer made up of 1,2-dipalmitoyl-sn-glycero-3-phosphocholine (DPPC), 1,2-dimyristoyl-sn-glycero-3-phospho-(1'-rac-glycerol) (DMPG), and cholesterol. Of many clinical trials performed with it worldwide, a phase IIb trial (NCT00157209) with patients with stage IIIB or IV NSCLC demonstrated an increment of 4.2 months in MS in the group receiving L-BLP25 compared to the cohort receiving BSC only. In a subset comprising patients with stage IIIB loco-regional NSCLC, an enhancement of 17.3 months in MS was observed, where the treatment group showed 49% 3-year survival compared to the control group with BSC showing 27% [266]. More light should be shed on the tailoring of patient-oriented cancer immunotherapy in concordance with the eventful and changeful dynamics of TME, which will help determine the timing and dosing of the therapeutic schedule. For instance, another phase III trial revealed that the liposomal tecemotide vaccine with concurrent chemoradiotherapy (NCT00409188) improved the OS to 9 months while sequential administration of chemoradiotherapy could not extend the OS, which hints at the importance of the timing of combinatorial therapy [267]. Table 3 summarizes a few clinical trials where the safety and efficacy of nanomaterials were evaluated for use in lung cancer immunotherapy.

**Table 3:** Clinical trials where the safety and efficacy of nanomaterials were evaluated for use in lung cancer immunotherapy

Clinical Trial ID	Drug	Delivery System	Cancer	Status	Duration	Sponsor/ Agency
NCT 02,049,151	Tecemotide Following Concurrent Chemo-radiotherapy	Liposome	Unresectable Stage III NSCLC	Phase III (USA)	2014–2015	EMD Serono/Oncothyreon Canada Inc
NCT 00,960,115	Tecemotide Following Chemotherapy	Liposome	Unresectable Stage III NSCLC	phase I/II (Japan)	2009–2015	Merck KGaA, Darmstadt, Germany
NCT 01,015,443	Tecemotide Following primary Chemo-radiotherapy	Liposome	Unresectable Stage III NSCLC	Phase III (Asian population)	2009–2015	Merck KGaA, Darmstadt, Germany
NCT 00,828,009	Tecemotide with bevacizumab after chemotherapy and radiation therapy	Liposome	Unresectable Stage IIIA/IIIB NSCLC	Phase II (USA)	2010–2019	ECOG-ACRIN Cancer Research Group
NCT 00,157,196	Tecemotide with single-dose low CPA	Liposome	Stage IIIA NSCLC	Phase II	2005–2012	Merck KGaA, Darmstadt, Germany
NCT 00,157,209	Tecemotide with single-dose low CPA	Liposome	Stage IIIB/Stage IV NSCLC	Phase IIB (Germany)	2000–2012	Merck KGaA, Darmstadt, Germany
NCT 00,409,188	Tecemotide with single-dose low CPA	Liposome	Unresectable Stage III NSCLC	Phase III (23 countries)	2007–2012	EMD Serono
NCT 03,836,352	DPX-Survivac with low dose Cyclophosphamide & Pembrolizumab	Liposome	NSCLC and other carcinomas	Phase II (USA/Canada)	2018–2023	ImmunoVaccine Technologies, Inc. (IMV Inc.)
NCT 00,291,473	Mixed cancer vaccines, CHP-HER2 and CHP-NY-ESO-1 with OK-432 (Picibanil)	Cholesterol-Bearing Hydrophobized Pullulan	Lung cancer and other carcinomas	Phase I (Japan)	2005–2008	Ludwig Institute for Cancer Research
NCT 01,853,878	Recombinant PRAME protein combined with the AS15 Adjuvant System GSK2302032A	Liposome	NSCLC after removal of the tumor	Phase II (9 countries)	2013–2016	GlaxoSmithKline
NCT 01,258,868	Tumor Cell Vaccines with ISCOMATRIX Adjuvant and Celecoxib	Liposome	Lung cancer and other carcinomas	Phase I (USA)	2010–2016	National Cancer Institute (NCI)

Few preclinical studies also elicited remarkable potential of nano-platforms for cancer immunotherapy. Interestingly, Moon and colleagues in 2017 synthesized a stable and homogeneous lipoprotein nanodisc, comprising phospholipids and 22 amino acid apolipoprotein mimetic peptides, to deliver neoantigen vaccines to draining lymph nodes. The nanodiscs evoked a strong anti-tumor T-cell response, eradicating established tumors and inhibiting metastatic tumor growth on murine lungs [268]. Alongside tissue and cellular targeting, molecular targeting has also shown great potential in cancer treatment, and their combinations with conventional chemotherapy have improved PFS in a phase III clinical trial with NSCLC patients having EGFR mutations [269]. It has also been proven now that the molecular targeting drugs can initiate immune responses via various mechanisms such as (i) aiding in antigen presentation by APC, (ii) instigating ICD in tumor cells, (iii) promoting T cell infiltration in TME, (iv) triggering NK cells, (v) attenuating the number of MDSCs, Treg, TAMs in TME. Nanotechnology and nanomaterials were used to improve molecularly targeted immunomodulation [270]. In 2020, Norvaline/Sunitinib encapsulating CuS photo-thermal nanoparticles were developed by Domvri et al. to target and exhaust MDSC subsets in lung TME. A549 tumor xenograft experiment revealed a marked anti-tumor effect simultaneously evoking strong innate and adaptive immune responses. It was shown that tumor infiltration of both CD8 + and CD4 + T cells was enhanced, and NK cells were activated with the diminution of MDSCs and Foxp3 + Treg cells (immune tolerance) [271]. A significant number of studies are ongoing to explore further the role of immunomodulatory nanomedicine in lung cancer immunotherapy, and we envisage an upsurge in their implications in the near future.

### Immunomodulatory nutraceuticals in lung cancer

In recent years, the use of nutraceuticals in the prevention and supportive care of cancer patients has drawn significant attention from researchers across the globe, and enormous efforts have been directed toward deciphering their therapeutic role and immunomodulatory mechanism of action in different cancers. For instance, the impact of curcumin had already been demonstrated by various groups in many tumor growth inhibition studies, which revealed its anti-cancer, anti-angiogenesis, and anti-metastatic properties. Recently, Zou et al. conducted a study with lung cancer patients, which showed that a two-week treatment with curcumin arrested the Treg cells and enhanced peripheral Th1 cells. Importantly, a conversion of Tregs to Th1 cells was observed via downregulating transcription of Foxp3 and upregulating the expression of IFN-γ, which can be attributed to curcumin treatment [272]. Even more recently, in a mechanistic investigation, dose-dependent regulation of PD-L1 expression by resveratrol was observed. At a lower concentration (< 5 μM), it induced PD-L1 expression in various lung cancer cells (H1299, A549, H460) via activating the Wnt pathway, but it inhibited PD-L1 expression at a higher dose (> 40 μM) [273]. Other nutraceuticals include apigenin, luteolin, phloretin, saponin, capsaicin, gallic acid, caffeic acid phenethyl ester, zerumbone, quercetin, etc. have also demonstrated their immunomodulatory potential against lung cancer. We provide a collection of evidence accumulated in recent years for immunomodulatory nutraceutical intervention in Table 4 [274–283]. It has been demonstrated that tumor-related inflammatory responses had the unanticipated effect of tumorigenesis and progression, assisting incipient abysmally grown tumors. Inflammation promotes neoplastic progression by generating an increasing number of signaling molecules, including EGF.

**Table 4:** Anti-proliferative, anti-inflammatory, and antioxidant potential of nutraceuticals against lung cancer

Target	Compound	Source	Bioactivity
**Lung Cancer**	Curcumin	Curcuma longa	Converted Foxp3 + regulatory T cells to Th1 cells in patients with lung cancer
	Resveratrol	grapes, wine, peanuts	Enhanced binding of β-catenin/TCF to PD-L1 promoter and increased PD-L1 expression. A higher dose (> 40 μM) resulted in a progressive reduction of PD-L1
	Apigenin	Parsley	Suppressed the translocation of NF-κB from the cytoplasm to the nucleus, which further inhibited target genes that block apoptosis
	Phloretin	Apples	Exerted anti-inflammatory activities on A549 cancer cell lines by reducing intercellular adhesion molecule 1 (ICAM-1) and cyclooxygenase (COX)-2 expression and suppressing monocyte adhesion through inhibition of MAPK, PI3K/Akt, and NF-κB signaling pathways
	Edible macro-fungi	beta-glucan	Significantly increased IFN-γ mRNA expression, increased M1 phenotype, and attenuated M2 phenotype of TAM
	Saponin	P. polyphylla Smith var. chinensis (Franch.) Hara	Decreased serum levels of TNF-α, IL-8, and IL-10, decreased expressions of proinflammatory cytokines MCP-1, IL-6, and TGF-β1
	Capsaicin	Chili	Inhibited the upregulation of TNF-α, IL-6, COX-2, and NF-κB expression
	Luteolin	Broccoli, Apple skin	Inhibited monocyte recruitment and cancer cell migration via suppression of the TAM-secreted CCL2
	Gallic acid	Grape seeds, rose flowers, sumac, oak, and witch hazel	Suppressed LPS-induced NF-κB activation in A549 lung cancer cells, inhibited IFN-γ, IL-6, IL-1β & NF-κB regulated anti-apoptotic genes expression
	Caffeic acid phenethyl ester	Propolis	Inhibited NF-kB, caffeic acid phenethyl ester significantly diminished the induction of inflammatory gene
	Zerumbone	Zingiber officinalis	Inhibited lung carcinogenesis by modulating the expression of NF-κB and heme oxygenase-1
	Quercetin	Grapes, Berries	Suppressed the nuclear translocation of NF-κB and reduced levels of inflammatory cytokine TNF-α, IL-1, and IL-6, significantly increasing the NK-cell-mediated cytotoxic activity against lung cancer cells
	EPA, DHA, and Se	Fish oil and Selenium yeast	Synergistically decreased the population of splenic Tregs and MDSCs and thus augmented host anti-tumor immunity against lung carcinoma

It has been elicited that tumor-related inflammatory responses had the unanticipated effect of exacerbating tumorigenesis and progression, helping incipient abysmally grown tumors acquire cancer-specific salient characteristics and promote their impending development into full-blown cancers. Inflammation contributes to neoplastic growth by providing a growing list of signaling molecules: EGF, VEGF, FGF2, chemokines, cytokines that promote the inflammatory state, and matrix-degrading MMPs, cysteine cathepsin proteases, heparanase, and inductive signals that lead to activation of epithelial-mesenchymal transition (EMT), etc. Moreover, inflammatory cells secrete chemicals, particularly reactive oxygen species (ROS), which are proactively mutagenic for neighboring tumor cells, facilitating their genetic evolution.

oxidant properties have extensively been used in research to explore their anti-cancer, anti-angiogenesis, and anti-metastatic properties [284]. Anti-inflammatory agents have increasingly been used as effective adjuvants for conventional therapies, and three mechanisms have been proposed—(i) chemo-protection, (ii) alterations in pharmacokinetics or metabolism, and (iii) chemo-sensitization [285, 286]. Notably, a study by Menendez and colleagues in 2016 revealed that oral intake of silibinin significantly inhibited brain metastasis of NSCLC patients. Marked tumor growth inhibition was also shown in NSCLC xenograft by oral gavage of silibinin [287]. Taken together, therapeutic immunomodulatory nutraceutical intervention has conferred several benefits in lung cancer in both preclinical and clinical settings.

### Ongoing clinical trials for immunotherapeutics

Different clinical trials are ongoing to assess various existing and new immunotherapeutic agents developed worldwide to assess their application in the management of lung cancer. A few recent trials conducted on NSCLC and SCLC patients are mentioned in Tables 5 and 6 [242, 243]. It is to be noted that the important phase II/III trials whose results have been analyzed have already been discussed in the previous sections. A few important ongoing trials are discussed below.

**Table 5:** Ongoing randomized phase II and phase III Trials in Early and Locally Advanced NSCLC

Neoadjuvant Strategy	Trial Identifier (NCT reference)	Phase	Drug/Treatment
IB-IIIA	CheckMate 816 (NCT 02,998,528)	III	Nivo + Ipi vs. chemotherapy
IIIA	(NCT03081689)	II	Nivo + chemotherapy
IB (> 3 cm)-IIIA	TOP 1501 (NCT02818920)	II	Pembro
IB (> 4 cm)-IIIA	PRINCEPS (NCT02994576)	II	Atezo
IB-IIIA	MAC (NCT02716038)	II	Atezo + chemotherapy
I (> 2 cm)-IIIA	(NCT02904954)	II	Durva or Durva + SRBT
IB(> 4 cm)-IIIA	ANVIL (NCT 02,595,944)	III	Nivo vs Obs
IB(> 4 cm)-IIIA	PEARLS(NCT02504372)	III	Pembro vs. placebo
IB(> 4 cm)-IIIA	IMpower010(NCT02486718)	III	Atezo vs. BSC
IB(> 4 cm)-IIIA	BR-31(NCT02273375)	III	Durva vs. placebo
Unresectable III A/B	RTOG3505(NCT02768558)	III	CRT Nivo vs. placebo
Early stage unresected NSCLC	NCT03833154	III	Durvalumab + SBRT vs placebo + SBRT vs Osimertinib + SBRT
IIA- IIIB NSCLC	ALCHEMIST (NCT04267848)	III	Pembrolizumab + chemotherapy
Early stage NSCLC	CANOPY-N(NCT03968419)	II	Canakinumab ± Pembro
III-IV NSCLC	NCT03793179	III	Pembro ± chemotherapy
III-IV NSCLC	IGNYTE(NCT03767348)	II	RP 1 ± Nivolumab
PD-L1 expressing NSCLC	NCT04432207	I	IMU-201 (PD1-Vaxx)
III-IV NSCLC	NCT04007744	I	Pembro + Sonidegib

Abbreviations: *Nivo* Nivolumab, *Ipi* ipilimumab, *Pembro* Pembrolizumab, *Atezo* Atezolizumab, *Durva* Durvalumab, *SBRT* Stereotactic body radiotherapy, *Obs* Observation, *BSC* Best supportive care, *CRT* chemoradiotherapy

**Table 6:** Ongoing Clinical Trials Investigating Immunotherapy in SCLC

Trial Identifier (NCT reference)	Drug/Target	Phase	Treatment Regimen
**Limited stage**			
NCT02402920	Pembro/PD-1	I	MK-3475 + PE + Thoracic RT
NCT02934503	Pembro/PD-1	II	Pembro + PE + Thoracic RT
**Extensive stage**			
NCT03066778	Pembro/PD-1	III	Pembro + PE vs Placebo + PE
NCT02763579	Atezo/PD-L1	I/II	Atezo + Carboplatin + Etoposide Vs. Placebo + Carboplatin + Etoposide
**Extensive stage-Maintenance**			
NCT03043599	Ipi Nivo/CTLA-4 PD-1	I/II	Consolidative Ipi + Nivo and maintenance Nivo with Thoracic RT after platinum-based chemotherapy
RAPTOR (NCT04402788)	Atezo	II/III	Atezo vs Atezo + RT
**Extensive stage-Second-line and beyond**			
NCT03026166	Ipi Nivo/CTLA-4 PD-1	I/II	Rova-T and Nivo or Rova-T and Nivo plus Ipi 1 mg/kg or Rova-T and Nivo plus Ipi 3 mg/kg

Abbreviations: *Pembro* Pembrolizumab, *PE* platinum/etoposide, *Rt* Radiotherapy, *Atezo* Atezolizumab, *PD-1* Programmed cell death-1, *Ipi* ipilimumab, *Nivo* Nivolumab, *CTLA-4* cytotoxic T-lymphocyte antigen, *Rova-T* Rovalpituzumab tesirine, *Durva* Durvalumab

CheckMate 816 is a phase III trial among 326 stage IB-IIIA NSCLC patients for comparing treatment outcomes with Nivolumab and Ipilimumab versus standard chemotherapy. PEARLS is a phase III trial in stage IB-IIIA of 1380 NSCLC patients comparing DFS between Pembrolizumab versus placebo. IMpower010, a phase III trial in stage IB-III A of 1127 NSCLC patients, is directed to assess the difference in DFS between Atezolizumab and best supportive care. Another crucial ongoing phase III trial of unresectable III A/B, 660 NSCLC patients is RTOG3505 and aims to compare OS and PFS after chemotherapy followed by Nivolumab versus placebo. Another phase III ALCHEMIST clinical trial (ANVIL) examines the effect of adjuvant Nivolumab in OS and/or DFS over standard observation after surgery and standard adjuvant therapy in 714 stage IB-IIIA NSCLC patients. The other notable phase III trial is NCT03066778, in 430 extensive-stage SCLC patients expressing PD-1 compares PFS with Pembrolizumab and Platinum/Etoposide versus placebo and Platinum/Etoposide. In another ongoing phase II/III trial (RAPTOR) with extensive-stage SCLC patients in maintenance therapy,138 patients in phase II, and 186 patients in phase III are compared for PFS with Atezolizumab alone versus Atezolizumab and radiotherapy. While NCT02934503: This is a phase II trial on 60 extensive stages SCLC patients assessing progression-free survival with Pembrolizumab, Platinum/Etoposide, and Radiotherapy.

### Major obstacle and future perspective for lung cancer treatment

Immunotherapy has shown tremendous promise in lung cancer therapy but is still in its infancy. A significant hurdle is the absence of optimal appropriately standardized in vitro and in vivo laboratory-based, preclinical, and clinical model systems in evaluating the efficacy, mechanism, kinetics, and toxicity of immunotherapy and immune modulators [288]. Immunocompetent mouse-in-mouse models are often used, including the genetically engineered mouse models (GEMMs), chemically induced models, and syngeneic tumor graft models [289, 290]. Though existing models address some aspects, they do not provide the complete coverage needed to understand the basic mechanisms of immune biology and to evaluate new immunotherapies. Humanized mice, or human immune system (HIS) mice, which have both the human immune system and human tumors, are increasingly employed in preclinical immunotherapy investigations but are extremely costly [291]. Organoids or tumoroid with immune cell 3D co-culture models and microfluidic-based organoids-on-a-chip models are developed to use patient-derived tumor cells. These technologies have great potential but are still in the early stage of development. Thus, there is an urgent need to develop trustworthy models for understanding the dominant drivers of cancer immunity, immune mechanism, therapeutics, primary and secondary immune escape, synthetic immunity, and toxicity studies for translational cancer immunotherapy.

Further research is essential to understand better different immune aspects of lung cancer, including immune escape, immunosuppression, immune editing, and tumor-intrinsic adaptive response to immunotherapeutic stress, to reactivate and reliably channel the patient's immunity against cancer. Combination immunotherapy is effective, but there is no consensus on selecting the treatment strategy. Future NSCLC therapies will possibly comprise a combination of chemotherapy, neoantigen vaccinations, and several ICIs to target the rewired tumor signaling pathways. In combination with ipilimumab, nivolumab is the most potential immunotherapy cocktail for advanced NSCLC patients. The prospect of different classes of novel immune modulators in combination immunotherapy is almost unexplored. The safety, tolerability, and effectiveness of monotherapy and combination immunotherapeutics are now being studied in clinical trials. Epigenetic alterations in a complex interaction with genetic alterations lead to lung tumorigenesis. Changes in the epigenetic landscape can cause dysregulation of oncogenes and tumor suppressor genes, leading to heightened proliferation, faster cell cycles, apoptosis resistance, and immune modulation [292]. Epigenetic therapy is mainly focused on DNA methyltransferase inhibitors (DNMTi), histone deacetylase inhibitors (HDACi), Janus kinase 2 inhibitors and RNA-based therapeutics in combination with immunotherapeutics can boost the effectiveness of current lung cancer therapies for long-term patient survival [293]. Co-targeting tumor-intrinsic and tumor ecosystem-associated adaptive stress pathways, including metabolic, oxidative, endoplasmic reticulum, DNA damage, and replication stress response, can help design translatable combination therapies. Immunotherapeutics can be combined with epigenetic inhibitors (e.g., DNMTi, HDACi, EZH2i) to target epigenetics-mediated tumorigenesis and immunosuppression.

The advent of bulk and single-cell multi-omics studies has paved the way to evaluate and customize immunotherapy at a personalized level. Each patient's tumor is unique, and the collection of all somatic cancer mutations found in a single tumor is termed ‘mutanome’. Lung cancer and smoking are interlinked and may exhibit smoking-associated neoepitope signatures. Personalized and unique lung cancer-specific neoantigens can be detected and.

targeted by ACT therapy. The ACT arsenal for lung cancer treatment is expanding. CAR T, TCR, and TIL therapy have made significant clinical inroads, but several challenges exist and are discussed in the respective section. Chemokines influence T cell recruitment and infiltration into lung tumors, and a higher CD4 + and CD8 + T lymphocyte infiltration is a favorable prognostic indicator [294]. Jin et al. recently used CCR6-expressing CAR T cells to target lung cancer using a xenograft mouse model and showed promising T cell infiltration and tumor killing [295]. Earlier Adachi et al. engineered CAR-T cells to express IL-7 and CCL19 (crucial for maintaining T-cell zones in lymphoid organs) and showed promising results with Lewis Lung carcinoma in a mouse model [296]. Additionally, improvements in multi-omic platforms at a single cell level and access to publicly available data sets can help better apprehend the immune landscape for educated therapeutic selection.

A personalized immunotherapy approach needs to be investigated in lung cancer. Tumor heterogeneity can be a significant bottleneck in designing personalized immunotherapies. The immune microenvironment is dynamic, and spatiotemporal analysis of different types of lung cancer can help understand the immune repertoire, antigen-presentation modes, and immune editing. Recent developments in multi-spectral analysis techniques, like multiplex immunofluorescence (MIF), Imaging mass cytometry (IMC), Chipcytometry, Multiplexed Ion Beam Imaging (MIBI), DNA barcoding-based mIHC/IF, and insitu-plex can help in deciphering the tumor immune repertoire [297]. Whole exome sequencing (WES), RNA-seq, single-cell sequencing, and TCR sequencing can reveal the tumor mutanome and cell-specific TCRs that are neoantigen-specific and aid in the cancer vaccination approach [298]. Several factors like TCR diversity, TCR degeneracy, neoantigen clonality, neoantigen subtype, and differential agretopicity index offer challenges. Each patient's cancer cells have a unique cocktail of neoantigen–MHC complexes (termed the neoantigenome). Multiple AI-based multi-component computational algorithms can examine the binding complementarity of the mutant peptide and the patient's HLA alleles and evaluate the potential to develop an anti-tumor T cell response. AI approaches like TSNAD, pVAC-Seq, INTEGRATE-neo, NetMHCpan, MARIA, EDGE, and DeepHLApan employ multi-layer architecture to find patterns and predict MHC-I/II binding and neoantigen binding efficacy and immunogenicity [299–301]. Though in silico binding prediction can yield helpful information, LC–MS/MS analysis of MHC molecules immunoprecipitation and peptide identification will produce an accurate and robust database, e.g., The Immune Epitope Database (IEDB) [301]. Personalized neoantigen identification can help develop next-generation immunotherapeutics. For preclinical and clinical applications, the binding affinity of the neoantigen to the corresponding MHC is predicted, and affinities greater than 500 nM are considered immunogenic neoepitopes and subsequently selected for the development of customized cancer vaccines [302]. There is an urgent need to discover lung cancer-specific composite biomarkers for categorizing tumor immunogenicity, patient stratification, pharmacodynamic prediction, and finalizing regulatory endpoint to improve lung cancer immunotherapy. Recent advances in AI-based algorithms and models will soon be able to predict immunotherapy and combination therapy responses at a personalized level to further the efficacious use of immunotherapy at a clinical level [303].

Though immunotherapy incites immunememory and is the most promising way to treat cancer and is better tolerated with minimum side effects as per clinical data, some patients suffer from immune-related adverse effects (irAEs) [304]. The side effects include flu-like symptoms, skin rash, pain, edema, heart palpitations, diarrhea, an overly activated immune status, and damaging organ systems. Cytokine release syndrome and onset of diabetes are also observed in CAR-T and immunotherapy patients. Interstitial and alveolar infiltrates followed by pneumonitis is the most common immune-related adverse event in the lung [305]. Though immunosuppressive corticosteroids are the choice for treating irAEs but are also reported to reduce the efficacy of the immunotherapy. Studies regarding immunomodulatory nanomaterials and nutraceuticals may open up new avenues in addressing irAEs and autoimmune toxicities. Additional research is required to comprehend the process of irAE better to manage the adverse effects of lung cancer immunotherapy.

## Conclusion

With the discovery of immunotherapy, the therapeutic paradigm for patients with advanced lung cancer has fundamentally transformed lung cancer treatment and is still evolving. ICIs have improved patient OS while causing fewer adverse effects than traditional chemotherapeutic medicines and have become an integral part of treatment algorithms. Several possible therapeutic options are available for advanced-stage lung cancer patients, from single-agent immunotherapy to quadruple therapy, which combines immunotherapy with chemotherapy and anti-vascular endothelial growth factor medications. In order to treat advanced lung cancer patients, the U.S. FDA has approved immunotherapy medications alone or in combination with other immunotherapies and chemotherapy, as reviewed in this article. Generation of resistance to ICIs, whether intrinsic or acquired, is a significant issue for the oncology community. Cellular therapy is a potential and practical addition to the arsenal of lung cancer immunotherapies. Due to the absence of tumor-specific antigens, a hostile TME, and toxicity, cellular treatment is an attractive but unquestionably challenging prospect. Clinical studies evaluate innovative therapeutic methods, including the combination and sequencing of PD-1/PD-L1 inhibitors with different ICIs and DNA repair targeting medicines. Overall, the therapeutic advantages of ICIs and ACT have exhibited promising trends for efficacious lung cancer therapy in the future.

## Data Availability

Not applicable.

## Abbreviations

PAH: Polycyclic aromatic hydrocarbons
WHO: World Health Organization
NSCLC: Non-small cell lung cancer
SCLC: Small cell lung cancer
LUAD: Lung adenocarcinoma
LUSC: Lung squamous cell carcinoma
LCC: Large cell carcinoma
LDCT: Low-dose computed tomography
LD-SCLC: Limited disease SCLC
ED-SCLC: Extensive disease SCLC
MS: Median survival
TSA: Tumor-specific antigen
TAA: Tumor-associated antigens
NK: Natural killer cells
DC: Dendritic cells
MΦ: Macrophages
APC: Antigen presenting cell
mAb: Monoclonal antibody
MHC: Major histocompatibility complex
IASLC: International Association for the study of Lung Cancer
CKI: Checkpoint inhibitor
PD-1: Programmed cell death-1
PD-L1: Programmed death-ligand 1
FDA: U.S. Food and Drug Administration
EGF: Epidermal growth factor
BSC: Best supportive care
CTA: Cancer testis antigen
NY-ESO-1: New York esophageal squamous cell carcinoma 1
MAGE-A3: Melanoma-associated antigen-A3
DFS: Disease-free survival
MUC-1: Mucin 1
MVA: Modified Vaccinia Virus Ankara
TGF-β2: Transforming growth factor β2
CTL: Cytotoxic T lymphocytes
ACT: Adoptive cell therapy
TCR: T-cell receptor
TIL: Tumor-infiltrating lymphocytes
CAR-T: Chimeric antigen receptor-modified T cells
CEA: Carcinoembryonic antigen
MSLN: Mesothelin
HER2: Human epidermal growth factor receptor 2
GPC3: Glypican-3
CAR T: Chimeric antigen receptor (CAR)-modified T cells
TIL: Tumor-infiltrating lymphocyte
TCR: T-cell receptor
HLA: Human leukocyte antigen
TMB: Tumor mutational burden
BiTEs: Bispecific T-cell engagers
ROR1: Receptor tyrosine kinase-like orphan receptor
DNT: Double-negative T cells
OV: Oncolytic Virus
HSV-1: Herpes Simplex Virus-1
CVB3: Coxsackievirus B3
ICD: Immunogenic cell death
OVV: Oncolytic vaccinia viruses
SVV-001: Seneca Valley Virus isolate 001
ADC: Antibody–drug conjugate
BiTE: Bispecific antibodies
irPFS: Immune-related progression-free survival
OR: Objective response
AE: Adverse event
TIGIT: T cell immunoreceptor with Ig and ITIM domains
BTD: Breakthrough therapy designation
ORR: Overall response rate
PPV: Personalized peptide vaccination
TMB: Tumor mutational burden
LEMS: Lambert-Eaton myasthenic syndrome
EMT: Epithelial-mesenchymal transition
ROS: Reactive oxygen species
GEMM: Genetically engineered mouse models
HIS-mice: Human immune system mice
MPL: 3-O-Deacyl-4’-monophosphoryl lipid A
DPPC: 1,2-Dipalmitoyl-sn-glycero-3-phosphocholine
DMPG: 1,2-Dimyristoyl-sn-glycero-3-phospho-(1'-rac-glycerol)
MDSC: Myeloid-derived suppressor cells
Tregs: Regulatory T cells
